# Saliva and Serum Cytokine Profiles During Oral Ulceration in Behçet’s Disease

**DOI:** 10.3389/fimmu.2021.724900

**Published:** 2021-12-22

**Authors:** Tanya Novak, Mojgan Hamedi, Lesley Ann Bergmeier, Farida Fortune, Eleni Hagi-Pavli

**Affiliations:** ^1^ Department of Anesthesiology, Critical Care and Pain Medicine, Boston Children’s Hospital and Department of Anesthesia, Harvard Medical School, Boston, MA, United States; ^2^ Centre for Oral Immunobiology and Regenerative Medicine, Barts and The London School, of Medicine and Dentistry, Queen Mary University of London, London, United Kingdom

**Keywords:** Behçet’s disease, cytokines, saliva, ulceration, oral mucosa, immune profiling, inflammation, FlowCytoMix

## Abstract

Behçet’s disease (BD) is a chronic, multi-systemic disorder of unknown aetiology typified by recurrent oral and genital mucocutaneous lesions, uveitis and vasculitis. Innate and adaptive immune system dysregulation has been implicated in pathogenesis with alterations in serum cytokine profiles. Few studies have investigated salivary cytokines in BD, despite more than 90% of BD patients first presenting with oral ulceration. The aim of this pilot study was twofold; firstly to investigate whether cytokine levels in matched serum and saliva samples show a differential profile in BD (with and without oral ulcers), recurrent aphthous stomatitis (RAS) and healthy controls (HCs), and secondly, to explore if any differential profiles in serum and/or saliva could provide a panel of cytokines with diagnostic and therapeutic potential for BD. Concentrations of 12 cytokines (IL-1β, IL-2, IL-4, IL-5, IL-6, IL-8, IL-10, IL-12p70, IL-17A, IFN-γ, TNF-α, TNF-β) were measured using the Human Th1/Th2 11-Plex FlowCytomix™ kit with IL-17A, in BD (N=20), RAS (N=6) and HCs (N=10). A differential range of cytokines was detected in serum and saliva with the majority of cytokine levels higher in saliva. The most prevalent salivary cytokines were IL-1β, IL-2, IL-8, IL-10 and TNF-α present in all samples in contrast to serum where the most prevalent cytokine detected was IL-8 (91.9%). The least abundant cytokine was IFN-γ in both saliva (43.2%) and serum (2.7%). After normalizing saliva for protein content, BD patients with oral ulcers (BD-MA) had significantly higher levels of salivary IL-1β (p=0.01), IL-8 (p=0.02), TNF-α (p=0.004) and IL-6 (p=0.01) than HCs. Notably, BD patients without oral ulcers (BD-MQ) also had significantly higher salivary IL-1β, IL-8 and TNF-α (p ≤ 0.05) than HCs. During relapsed (BD-RE) and quiet (BD-Q) systemic episodes, salivary IL-β and TNF-α were also significantly increased with IL-8 significantly higher only in BD-Q (p=0.02). BD oral ulcers signify a potential reactivation of systemic inflammation. Identifying cytokines released during asymptomatic episodes and oral ulceration might lead to targeted drug therapy to prevent recurrent oral ulcers and possible disease relapse. This is the first study to report salivary cytokine levels in BD. The detectable levels suggests cytokine profiling of BD saliva may provide an alternative, less invasive, sensitive procedure for frequent monitoring of disease activity and progression.

## Introduction

Behçet’s disease (BD) is a chronic, multisystemic, recurrent vasculitis disease of unknown aetiology (1–3). Typified by early presentation with recurrent oral ulceration, patients may go on to present with genital ulcers, cutaneous lesions including erythema nodosum and folliculitis, joint involvement and life threatening vasculitis (4) as well as central nervous system disease such as meningo-encephalitis and neuro-psychiatric symptoms, although peripheral nerve/muscle damage is rare (5). Uveitis is also common and can lead to sight loss (6–10).

The disease is difficult to diagnose and in the absence of a definitive laboratory test, a differential clinical scoring system remains the only method to ensure correct diagnosis. First developed by the International Study Group for BD (11) it has since been ratified in an international 27 country survey (12). A definitive diagnosis may take many years to establish with concomitant impact on the quality of life of patients (13, 14).

Aetiopathogenesis remains unknown, but a common consensus suggests it may be triggered by an infection or an environmental stimulus in a genetically susceptible host (15, 16). Attempts to classify BD as an autoimmune, auto-inflammatory or a spondyloarthropathy fail as BD patients frequently exhibit features common to all these conditions (17, 18).

Infectious aetiology theories have indeed provided evidence of reactivity to Herpes Simplex (8), *Streptococcus sanguis* (19) and microbial heat-shock proteins (HSP) causing cross reactivity reactions with self-proteins as possible triggers of BD (20, 21). Cross reactivity of microbial HSP with self-proteins has been implicated in both BD and RAS but with very different outcomes (22). The ability to detect and respond to infection may also be impaired in BD where unusual splice variants of TLR2 and TLR4 suggest a defect in the crosstalk between innate and adaptive immune responses, and where significant reductions in the response to cognate agonists of TLR1/2 heterodimers have been observed in buccal cells (23).

Genetic susceptibility has also been investigated and strong associations with some HLA genes such as HLA-B51, a splice variant of HLA-B5, has been long established for multiple populations (8, 24–27). Several other studies showed links between BD and MHC class I chain related genes (MIC-A and MIC-B), also suggesting these antigens as candidates for genetic susceptibility as they are expressed on fibroblasts, gastric epithelium and endothelium cells and act as ligands for the NKG2D activating NK receptor found on both gamma delta (γδ) T cells and CD8^+^αβ T cells. Both of which have been found to have roles in BD pathogenesis, while NK cells have been found to be depleted in the circulation of BD patients (28–30).

More recently genome wide association studies (GWAS) have confirmed the association with HLA-B51 but have also pointed to associations with IL-10 variants, and variants lying between the IL-23 receptor (IL-23R) and IL-12 receptor (IL-12Rβ2) as well as IL-12A genes (31, 32). IL-10 is a potent suppressor of inflammation while IL-23 is a pro-inflammatory cytokine that stimulates T helper cell proliferation and increases the production of inflammatory cytokines such as IL-1, IL-6, IL-17 and TNF-α. These genes are engaged in both innate and adaptive immune response communication networks through cytokine signalling and support a hypothesis of immune dysregulation in BD (25, 27). Hyperfunctional neutrophils are characteristic of BD leading to an overactive neutrophil response (33, 34). Various neutrophil priming and activating factors are up-regulated in BD e.g. IL-8, TNF-α (35) and IL-1β (36). Furthermore, significantly increased levels of neutrophil elastase in plasma (37) and saliva (38) have been detected in both quiescent and active symptomatic BD. More recently a cell atlas of the oral mucosa has suggested a strong neutrophil/stromal cell regulatory interaction controlling tissue immunity (39).

One of the key immunological features of BD is alteration of blood cytokine levels (40, 41). Early work suggested that BD displayed a Th1 profile (42–44), and more recently Th1/Th17 cytokine polarisation of CD4^+^ T cells and increased IFN-γ, TNF-α, IL-8 and IL-17 levels have been correlated with BD activity (27, 44–46). However, cytokine production is often transient and tightly regulated, due to their high biological activity and the network within which they are up- or down-regulated according to need. Elevated cytokine levels in body fluids reflect activation of pathways associated with inflammation or disease progression in many disorders and as such offer a window into treatment regimens and/or disease progression monitoring (47, 48). The triggers for cytokine induction are not well understood and/or controversial and the master controllers for induction include an array of transcription factors able to cross activate or inhibit cytokine production. The Suppressor of Cytokine signalling (SOCS) proteins negatively regulate the JAK-STAT pathway of cytokine induction and have been found to be differentially expressed in BD patients. Comparisons made between expression of SOCS 1 and 3 mRNA in BD, RAS and HC showed an upregulation of these molecules in BD. Furthermore, there was a differential expression between buccal mucosal cells and peripheral blood cells which suggested that cytokines are differently regulated in the mucosa compared with the peripheral blood cells (monocytes and neutrophils) (49, 50).

While there have been many studies reporting differences in serum cytokine levels there have been no studies investigating levels in saliva. This is especially surprising considering between 85-100% of BD patients first present with oral ulceration which precedes the onset of all other symptoms and continue to erupt episodically (51). The oral microbial environment has been implicated in the pathogenesis of BD (16, 52) with patients experiencing new onset oral ulceration following dental and periodontal treatment (53, 54). Studies, including our own, have concluded that the oral health of BD patients is impaired (14, 55–57) while clinical treatment of oral symptoms results in better outcomes of systemic disease (38). Furthermore, the importance of dental and periodontal treatment for controlling oral ulceration activity and systemic disease has been reported (54) and highlighted in several studies, however its importance in the preventative management of BD has remained largely unrecognised (58).

BD and recurrent aphthous stomatitis (RAS) patients share nearly indistinguishable histological features of episodic oral ulceration (59), presenting with minor, major or herpetiform lesions. In BD, ulcers are more frequent and slower to heal sometimes with scarring not seen in RAS. Their oral biopsies are mainly described as non-specific with large infiltrations of neutrophils, some macrophages and mast cells (18, 60, 61). Gamma-delta (γδ) T cells may also be present which are rarely seen in normal oral mucosa (62, 63). Both BD and RAS ulcers are also positive for CD4 and CD8T cells as well as Th-1 cytokines such as IL-12, IFN-γ and TNF-α. However, Th-2 cytokines such as IL-4 have only been detected in BD ulcers (64). In the past, ELISA was the “Gold Standard” for cytokine analysis but in more recent times the development of multiplex assays has marked a step change in the ability to detect and measure multiple analytes in a variety of samples including serum, saliva, synovial and vitreous fluids (65–67). Furthermore, multiplex analysis allows the simultaneous measurement of many different proteins in smaller sample volumes and offers the ability to investigate multiple inflammatory networks in a single clinical sample (67).

Cytokines involved in pathogenesis of BD can be categorized into proinflammatory cytokines and chemokines, Th1 type, Th2 type and Th17 type cytokines. Therefore, we decided to measure 12 inflammatory cytokines and chemokines, representative of these categories, namely, IL-1β, IL-2, IL-4, IL-5, IL-6, IL-8, IL-10, IL-12 p70. IL-17A, IFN-γ, TNF-α and TNF-β in matched saliva and serum from BD, RAS, and HC. Firstly the aim was to investigate whether the levels and types of inflammatory cytokines measured in saliva reflect those in serum; and secondly, to characterize the cytokine profile of BD and RAS patients with and without oral ulcerations and/or systemic manifestations. For BD patients whose vasculitis makes drawing blood for routine tests a considerable challenge, saliva is more readily accessible and a less invasive window into their disease activity and progression.

## Materials and Methods

### Patients and Healthy Controls

Behçet’s Disease (BD) patients were recruited from outpatients attending the Behçet’s Centre of Excellence clinics at the Royal London Hospital, Bart’s and The London NHS trust that had been previously diagnosed according to the International Study Group for Behçet’s Disease (1990) with disease activity recorded using the ISBD activity form 2006. Individuals with recurrent aphthous stomatitis (RAS) were allocated as a disease control group and healthy controls were from self-identified healthy volunteers (HC). Matched serum and saliva samples were collected on the same day from 20 BD patients (7 males (M):13 females (F), mean age 38 ± 10.4 years, 10 HCs (5M:5F, mean age 34.7 ± 11.1 years) and RAS disease controls (matched RAS N=6, 4M:2F, mean age 38 ± 16. One RAS saliva and one RAS serum sample were from different patients. For RAS serum, 4M:3F, mean age 39.3 ± 15. For RAS saliva, 5M:2F, mean age 36.7 ± 15 years). The demographics for the patients and controls are described in **Tables 1A** and **B**, respectively. For this pilot study, if one or more oral ulcers were present during specimen collection, then the patient was deemed mouth active (BD-MA). If there was no mouth ulcer present, patients were recorded as mouth quiet (BD-MQ). BD patients were also assessed on their systemic activity during clinical attendance and deemed systemic active, or having relapsed, if they had at least three symptoms characteristic of BD according to the ISG (1990). The presence of activity in three or more clinical sites was considered as disease relapsed (BD-RE) and if not, disease quiet (BD-Q). BD medications are shown in **Table 1A**. Patients taking biologics were excluded from the study. The study was approved by the local research ethics committee (REC number P/03/122) and written informed consent was obtained from all patients and HC.

**Table 1A:** Behçet’s Disease Patients demographics for saliva and serum analysis.

Demographic and Clinical Features of Behcet’s Patients (N=20)		
Mean Age ±SD, Years	37.3±9.25	
Gender	Number (N)	%
Male	7	35
Female	13	65
Clinical Features		
Oral Ulcers	9	45
Genital Ulcers	4	20
Eye Lesions	3	15
Skin Lesions	3	15
Joint Involvement	9	45
Erythema Nodosum	2	10
Vascular Involvement	1	5
CNS	2	10
Relapsed	7	35
Treatment		
Prednisolone (PRED)	4	20
Azathioprine (AZA)	2	10
Colchicine (COLC)	3	15
PRED + AZA	2	10
PRED + COLC	1	5
PRED + COLC + AZA	3	15
AZA + COLC	1	5
Cyclosporine A + COLC	1	5
No Treatment	3	15

**Table 1B:** Demographics for Healthy Control (HC) and Recurrent Aphthous Stomatitis (RAS) study groups.

Demographics	Healthy Controls (N=10)		Recurrent Aphthous Stomatitis (RAS) N=7 (6*)					
			SERUM		SALIVA		MATCHED*	
Mean Age ± SD, Years	34.7±11.1		39.3±15		36.7±15		38±16	
Gender	N	%	N	%	N	%	N	%
Male	5	50	4	57.1	5	71.4	4	66.7
Female	5	50	3	42.9	2	28.6	2	33.3

Analysis was carried out on matched serum and saliva from all healthy control samples, however in the RAS cohort, *matched saliva and serum were collected on the same day for N=6 patients. One RAS serum and saliva sample were from different patients which is represented above in the different mean ages and gender numbers in the RAS serum and saliva patient cohorts.

### Serum Collection and Processing

Blood was collected into Vacutainers^®^ (Becton, Dickinson Co. UK) allowing coagulation in order to derive fibrinogen-free serum. Vacutainers^®^ were centrifuged at 3300 x g for 6 minutes (min) at room temperature (RT). Serum was aliquoted and immediately stored at -80°C and thawed immediately prior to cytokine analysis.

### Collection and Processing of Saliva Samples

Patients and HC volunteers rinsed with 5 ml of water prior to collection of unstimulated whole saliva and then asked to expectorate over a maximum period of 5 minutes into a 20 ml sterile universal tube that was then immediately placed on ice. From clinic, saliva samples were transferred to the laboratory, centrifuged at 4°C for 15 minutes at 3500 x g in order to remove cellular debris and saliva supernatants were then aliquoted and immediately stored at -80°C. Aliquots were thawed immediately prior to protein measurement or cytokine analysis. Total protein concentration was determined using the 2-D Quant Kit (GE Healthcare) as indicated by the manufacturer.

### Measuring Cytokines and Chemokines in Matched Saliva and Serum: FlowCytomix™ Multiplex Assay

The Human Th1/Th2 11-Plex FlowCytomix™ kit plus one extra cytokine, IL-17A (from eBioscience^®^ formerly Bender MedSystems^®^) was used to simultaneously measure a panel of 12 inflammatory cytokines and chemokines (IL-1β, IL-2, IL-4, IL-5, IL-6, IL-8, IL-10, IL-12 p70, IL-17A, IFN-γ, TNF-α and TNF-β) in undiluted, unstimulated saliva and matched undiluted serum from BD, RAS, and HC participants according to the manufacturer’s instructions. The limits and range of detection of the panel of cytokines provided by the manufacturer are shown in **Table 2**. A BD FACS Canto™ II flow cytometry instrument was used for the data collection. Analyte concentrations were calculated against the standard curves using the FlowCytomix™ Pro 3.0 Software.

**Table 2:** Number of samples with detectable cytokine levels in BD, RAS and HC matched saliva and serum as measured by the FlowCytomix assay.

	FlowCytoMix® detection limits pg/ml		Number of positive samples				Number of positive samples			
			Saliva				Serum			
	Lower	Upper	BD	RAS	HC	%	BD	RAS	HC	%
			N=20	N=7	N=10		N=19*	N=7**	N=10	
IL-1β	4.2	20,000	20/20	7/7	10/10	100	5/19	2/7	0/10	19.4
IL-2	16.4	20,000	20/20	7/7	10/10	100	11/19	4/7	5/10	55.6
IL-4	20.8	20,000	18/20	7/7	9/10	91.9	6/19	1/7	1/10	22.2
IL-5	1.6	20,000	19/20	7/7	10/10	97.3	5/19	2/7	2/10	25
IL-6	1.2	20,000	14/20	6/7	8/10	75.7	3/19	1/7	1/10	13.8
IL-8	0.5	10,000	20/20	7/7	10/10	100	19/19	6/7	9/10	94.4
IL-10	1.9	20,000	20/20	7/7	10/10	100	10/19	1/7	3/10	38.9
IL-12p70	1.5	20,000	20/20	6/7	10/10	97.3	4/19	0/7	2/10	16.7
IL-17A	2.5	10,000	18/20	7/7	8/10	89.2	3/19	1/7	3/10	19.4
IFN-γ	1.6	20,000	8/20	4/7	4/10	43.2	1/19	0/7	0/10	2.7
TNF-α	3.2	20,000	20/20	7/7	10/10	100	16/19	3/7	7/10	72.2
TNF-β	2.4	20,000	18/20	7/7	10/10	94.6	4/19	1/7	4/10	25

The lower and upper detection limits of the FlowCytomix assay are shown. Overall % prevalence for each cytokine in saliva (N=37) and serum (N=36).

*Denotes that one BD-MQ serum sample was excluded from the analysis due to an erroneous flow cytometer reading (BD N=19*). Therefore, *prevalence (%) out of 36 for serum samples.

**RAS cohort, matched saliva and serum was collected on the same day for N=6 patients. One RAS serum and saliva sample are each from different patients.

### Statistical Analysis

GraphPad Prism version 7.04 was used for analysis. Median cytokine measurements in each group were compared to one another using the Mann Whitney U, non-parametric statistical test (2 tailed). Significance is indicated by exact p values shown in figures and tables. Spearman’s Rho with two tailed significance was used for correlation analysis.

## Results

### Clinical and Demographic Characteristics

Of the 20 BD patients, investigated 65% were female and 35% male. BD relapse was diagnosed in 35% of the patients. The most frequent clinical symptoms at the time of sampling, were joint involvement and oral ulcers (45%) followed by genital ulcers (20%). Treatment information was available at the time of saliva and blood collection for all 20 BD samples; 17 patients were on immunosuppressive treatment (85%) with only three patients (15%) not receiving any medication (**Table 1A**).

### Comparison of Matched Saliva and Serum Levels in BD and RAS Patient Groups

The levels of Th1 cytokines, (IL-1β, IL-2, IL-12p70, IFN-γ, TNF-α and TNF-β), Th2 cytokines, (IL-4, IL-5, IL-6 and IL-10), chemokine IL-8 (CXCL8) and Th17 (IL-17A), were quantified in 36 matched serum and saliva samples from BD patients (**Table 1A**), HCs, and RAS (**Table 1B**).

**Figure 1:**
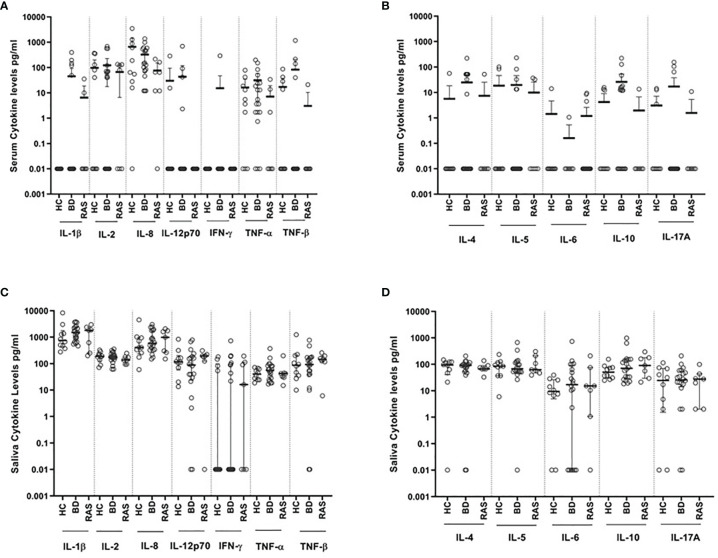
Levels of cytokines (pg/ml) detected in saliva and serum from BD (N=20), RAS (N=7) and HC (N=10). **(A)** Serum Th1 cytokines and IL-8 chemokine, **(B)** Serum Th2 cytokines and IL-17A, **(C)** Saliva Th1 cytokines and IL-8 chemokine, **(D)** Saliva Th2 cytokines and IL-17A.

Out of the 12 cytokines measured in saliva, the most prevalent were IL-1β, IL-2, IL-8, IL-10 and TNF-α being present in all 37 (100%) samples in contrast to serum where the most prevalent cytokine detected was IL-8, present in 94.4% of samples. The least abundant cytokine was IFN-γ in both saliva and serum, present in 45.9% and 2.7% of samples **(** **Figure 1**, **Table 2**), respectively.

**Figure 2:**
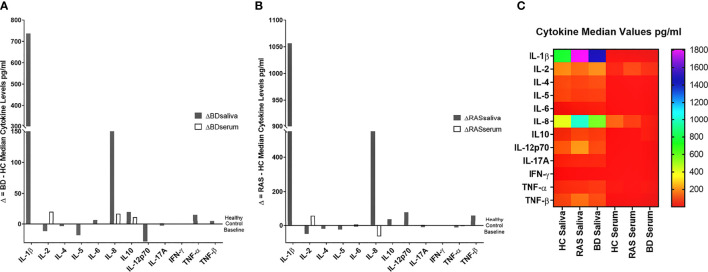
Group median cytokine differences between **(A)** BD and HC and **(B)** RAS and HC. For each cytokine, the following was calculated: Group Median BD cytokine concentration (pg/ml) - Group Median HC cytokine concentration (pg/ml) or Group Median RAS cytokine concentration (pg/ml) - Group Median HC cytokine concentration (pg/ml) to reveal the delta (Δ), or difference, of the disease group cytokine levels and the HC. **(C)** Heat map shows differential expression of 12 cytokines in saliva and serum.

Overall, when comparing cytokine values for the BD, RAS, and HC groups, there was a stark contrast in the prevalence of saliva cytokines compared to serum (**Figure 1**). This was also apparent when comparing the differences between BD and HC group median concentrations (ΔCytokine pg/ml) (**Figure 2** and **Supplementary Table S1**). For example IL-1β was measured in all BD saliva samples (median range 727.4 – 2383 pg/ml) (**Table 3**) but only in 19.4% of BD serum samples and with a lower median concentration (median range 0.01-36.8 pg/ml). This is the most marked increase followed by IL-8. In addition, An increase IL-6, TNF-α and TNF-β levels in BD saliva was evident with little or no change in serum levels relative to HC. IL-2 was higher in BD serum but lower in saliva, while IL-4, IL-5 and IL-12p70 were all lower in saliva with no marked changes to serum levels (**Supplementary Table S1**). IL-17A showed a small increase in saliva concentration compared with serum, whereas IL-10 levels were also low but similar in both saliva and serum when compared to HCs (**Figure 2**, **Table 3** and **Supplementary Table S1**).

**Table 3:** Concentrations of cytokines in serum and saliva of patients presenting with oral ulceration (Mouth Active (BD-MA)) or without (Mouth Quiet (BD-MQ)) oral ulceration involvement.

Cytokines	Group	Serum†		Saliva		p-values*
		Median	IQR	Median	IQR	
IL-1β	HC	0.01	0.01, 0.01	752.4	447.1, 1775	
	BD-MQ	0.01	0.01, 48.8	1168	933.7, 2233	0.22
	BD-MA	0.01	0.01, 85.2	1512	602.6, 3068	0.24
	BD-ALL	0.01	0.01, 36.8	1489	727.4, 2383	0.15
IL-2	HC	20.3	0.01, 185.1	187.8	125.4, 249.8	
	BD-MQ	0.01	0.01, 175.6	190.6	138.1, 238	0.85
	BD-MA	66.9	20.3, 96.8	173.6	137.7, 246.3	0.68
	BD-ALL	40.6	0.01, 71.3	176.4	139.6, 232.4	0.72
IL-4	HC	0.01	0.01, 0.01	94.8	40.5, 124.7	
	BD-MQ	0.01	0.01, 32.7	82.4	47, 116.6	0.74
	BD-MA	0.01	0.01, 51.9	93.1	67.6, 101.7	0.95
	BD-ALL	0.01	0.01, 51.9	91.4	53, 102.5	0.80
IL-5	HC	0.01	0.01, 21.2	85.9	36.9, 119.1	
	BD-MQ	0.01	0.01, 8.2	60.8	50.8, 114.2	0.59
	BD-MA	0.01	0.01, 13.5	95.2	46.9, 126.3	0.65
	BD-ALL	0.01	0.01, 13.5	67.8	51.3, 117.5	0.94
IL-6	HC	0.01	0.01, 0.01	9.5	4.9, 26.3	
	BD-MQ	0.01	0.01, 2.1	10.4	0, 18.9	0.69
	BD-MA	0.01	0.01, 0.01	63.9	13, 103.2	**0.04**
	BD-ALL	0.01	0.01, 0.01	17.1	0.01, 91.3	0.41
IL-8	HC	127.4	25.1, 975.5	410.3	237.5, 966.9	
	BD-MQ	135.8	65.9, 476.8	572.8	342.4, 1787	0.55
	BD-MA	363.9	44.5, 601.5	604.3	325.4, 1881	0.54
	BD-ALL	145	72.6, 535	588.5	329.6, 1804	0.47
IL-10	HC	0.01	0.01, 12.8	50.5	29.8, 78.4	
	BD-MQ	6	0.01, 41.4	75.7	26.9, 142.9	0.59
	BD-MA	13	0.01, 17.3	65.9	28, 160	0.67
	BD-ALL	11.9	0.01, 16.5	70.8	28.54	0.57
Il-12p70	HC	0.01	0.01, 3.9	116.5	57.8, 203.7	
	BD-MQ	0.01	0.01, 0.6	82	5, 161.4	0.39
	BD-MA	0.01	0.01, 21.1	94.8	34.7, 214.3	0.84
	BD-ALL	0.01	0.01, 0.01	88.4	23.4, 182.5	0.50
IL-17A	HC	0.01	0.01, 5.8	24.4	1.5, 70	
	BD-MQ	0.01	0.01, 16.1	27.2	12.7, 48	0.74
	BD-MA	0.01	0.01, 0.01	21	10.7, 59.2	0.86
	BD-ALL	0.01	0.01, 0.01	25.7	14, 55.2	0.75
IFN-γ	HC	0.01	0.01, 0.01	0.01	0.01, 98.8	
	BD-MQ	0.01	0.01, 0.01	0.01	0.01, 89.9	1
	BD-MA	0.01	0.01, 0.01	0.01	0.01, 147.8	0.75
	BD-ALL	0.01	0.01, 0.01	0.01	0.01, 93.5	0.86
TNF-α	HC	0.01	4.6, 24.6	40.2	24.4, 64.5	
	BD-MQ	1.3	23.9, 100.9	42.2	18.7, 81.5	0.96
	BD-MA	1.2	3.6, 13	59.0	40.4, 123.9	0.10
	BD-ALL	5.5	1.7, 24.4	55.8	25.4, 83.3	0.36
TNF-β	HC	0.01	0.01, 32.1	87.4	33.3, 209	
	BD-MQ	0.01	0.01, 35.6	53.9	38.3, 170.5	0.69
	BD-MA	0.01	0.01, 20.1	114.8	47.4, 163.2	0.74
	BD-ALL	0.01	0.01, 0.01	92.9	38.8, 169.5	0.96

†Serum concentrations showed no significant differences between groups.

*p-value when compared with HC saliva. Significant values in bold.

Serum HC N=10, BD-MQ N=10, BD-MA N=9, BD-ALL N=19. Non-normalized saliva HC N=10, BD-MQ N=11, BD-MA N=9, BD-ALL N=20. Median concentrations with interquartile ranges (IQR) are shown. Cytokines below the lower levels limits of detection were considered not detectable and therefore arbitrarily assigned concentrations of 0.01 pg/ml. p values are given for comparisons between HC and BD subgroups.

In RAS saliva and serum, IL-1β, IL-8, and TNF-β were also higher with no change in serum levels for IL-1β and TNF-β, however IL-8 serum levels were lower in RAS relative to HC. IL-2 levels were higher in RAS serum and decreased in saliva. Similarly, in BD saliva, IL-2, IL-4 and IL-5 were also decreased in RAS saliva. Conversely, IL-12p70 was increased in RAS saliva whereas in BD saliva, IL-12p70 was decreased relative to HCs.

Comparison of BD and RAS group median Δ cytokine levels also highlighted some key differences between these patient groups (**Figure 2**). For instance, IL-8 and IL-10 are increased in both BD saliva (ΔIL-8 = 178.2pg/ml, ΔIL-10 = 20.35pg/ml) and serum (ΔIL-8 = 17.6 pg/ml, ΔIL-10 = 11.9 pg/ml) whereas in RAS patients, IL-8 and IL-10 levels are higher only in saliva (ΔIL-8 = 575 pg/ml, ΔIL-10 = 40.3pg/ml) (**Supplementary Table S1**).

### Comparing Th1, Th2, and Th-17 Cytokine Levels in Serum and Saliva

Out of all the saliva Th1 cytokines, IL-1β and IL-8 were detected at the highest levels across all patient groups (**Table 3** and **Table S2**). IL-8 levels were higher in saliva than serum with the group median levels of salivary IL-8 in BD (MA and MQ) and RAS (MA and MQ: data not shown, RAS-ALL **Supplementary Table S2**) all higher than HCs however in serum, only BD IL-8 levels were higher (**Table 3**). IL-1β levels were the highest in saliva with both BD and RAS levels higher than HCs. Of all groups, BD-MA, had the greatest concentration however, this did not reach significance. In contrast, no IL-1β was detected in HC serum, with very low levels of IL-1β in RAS and only five of the 19 BD patients having measurable levels. It was interesting to note that the serum cytokine concentration range was markedly reduced compared to saliva; however, these differences did not reach significance among all the study groups. Maximum values obtained in a serum from a BD-MA sample was 1367 pg/ml (**Figure 1A**) whereas in saliva the range for all the groups was up to 4000 pg/ml with only one HC outlier sample giving a value of more than 8000 pg/ml (**Figure 1C**).

IL-2 was readily detected in all saliva samples but in only half of serum samples. Serum and saliva cytokine levels of Th1 cytokines IL-12p70 and IFN-γ did not reach any significant differences among all the study groups (**Table 3** and **Supplementary Figures S1, S2** respectively). Saliva IFN-γ was detected in only 16 out of 37 samples and was the least abundant cytokine in saliva.

Amongst the Th2 cytokine measurements for saliva (**Table 3**, **Supplementary Figure S2**), the patient groups had medians below 100 pg/ml. HC, BD-MQ, and BD-MA saliva had a similar IL-4 median concentration. In serum, IL-4 was detected in only 31.6% of BD samples and in one RAS and HC sample (**Table 2**). Similarly, IL-5 was detected in almost all saliva samples but only 25% of serum samples across all groups (**Table 2**). IL-5 HC median level of 85.9 pg/ml in saliva was higher than BD-ALL, BD-MQ and RAS 67.7, 60.8 pg/ml and 62.8 pg/ml respectively, however this did not reach significance (**Table 3** and **Supplementary Figure S2**).

IL-6 was detected in 13.8% serum samples but found in 75.7% of saliva samples across the cohort (**Table 2**). Median IL-6 concentrations were higher in BD and RAS patients compared to HCs, however only IL-6 reached a significant increase in BD-MA saliva compared with HC (p=0.04) (**Table 3**). Further analysis revealed that IL-6 levels were also significantly higher in the saliva of BD patients with ulcers (BD-MA) than those without (BD-MQ, p=0.03) (**Supplementary Figure S2**).

The data obtained for IL-17A showed that while it was detected in only a few serum samples, it was present in almost all saliva samples tested across all the groups however there was no significant differences (**Tables 2**, **3**).

### Cytokine Levels and Medications

Since most of the BD patients in this study were receiving immunosuppressive therapies (85%) at the time of saliva and serum collection, it was important to investigate if the treatment regimen affected cytokine concentrations. Therefore we compared saliva and serum samples (data not shown) from patients on single therapy (ST) [i.e. prednisolone (PRED) or azathioprine (AZA) or colchicine (COLC)-only medication (N=9)], or a combination of different therapies (CT) that included combinations of PRED, AZA, COLC and/or Cyclosporin A (CY-A) (N=8) with patients not on any medication (N=3). No medications were reported for RAS patients and HC groups.

All Th2 salivary cytokine levels were higher in BD patients receiving no treatment. IL-17A, IL-2, IL-12p70, IFN-γ, TNF-α and TNF-β were also higher whereas IL-1β and IL-8 levels were more than 2-fold lower in the BD patients not receiving any medication (**Table 4A**) but this did not reach statistical significance. Next, we wanted to investigate whether COLC and AZA alone or in combination affected salivary cytokine levels since we have previously reported that BD patients treated with both these medications have significantly lower neutrophil elastase levels (38) during episodes of oral ulceration. The general trend was that patients on neither COLC or AZA had slightly higher IL-1β, IL-2, IL-4, IL-5, IL-6, IL-12p70, IL-17A, IFN-γ, TNF-α and β saliva concentrations than BD patients on both AZA and COLC treatment however this was not significant (**Table 4B**). In contrast, IL-8 and IL-10 levels were lower in patients on neither medication, 572.8 pg/ml and 56.9 pg/ml as compared to patients on both COLC and AZA, 835.6 pg/ml and 70.8 pg/ml respectively. Of note, IFN-γ the least detectable cytokine in saliva, was still quantifiable in 43.2% of BD saliva samples and had a higher median in BD patients not receiving COLC, AZA or a combination of these medications (**Table 4B**).

**Table 4A:** Saliva cytokine concentrations from Behçet’s Disease (BD) patients on Single treatment (ST), Combined Treatment (CT) or No Medication (NO).

Cytokines pg/ml	Single Therapy (ST)	Combined Therapy (CT)	No Medication (NO)	Ratio ST/NO	Ratio CT/NO
	N=9	N=8	N=3		
IL-1β	**2433** (759.2-3665)	**1317** (958.6-2057)	**658.7** (546.5-1667)	3.7	2.0
IL-2	**173.6** (167.1-206)	**167.4** (94.2-296.9)	**276.9** (109.1-2867)	0.6	0.6
IL-4	**82.4** (51.7-96.5)	**94.8** (58.6-128.6)	**95.56** (38.9-131.1)	0.9	1.0
IL-5	**60.8** (51.8-86)	**77** (51.3-134.2)	**118.5** (26.6-670)	0.5	0.7
IL-6	**18.9** (1.2-103)	**13.3** (0.01-43.5)	**23.7** (0.01-136.6)	0.8	0.6
IL-8	**604.3** (372.3-2160)	**654.3** (285.5-910)	**325.4** (148.9-1787)	1.9	2.0
IL-10	**56.9** (24.7-147.7)	**70.8** (25.9-129.4)	**162.6** (33.6-983.1)	0.4	0.4
IL-12p70	**82** (19.7-178)	**67.8** (9.1-149.2)	**241.5** (29.9-786.8)	0.3	0.3
IL-17A	**27.2** (30.8-38.7)	**15.3** (0.5-67.3)	**57.6** (2-210.9)	0.5	0.3
IFN-γ	**0.01** (0.01-97.7)	**0.01** (0.01-141.6)	**43.3** (0.01-93.5)	0	0
TNF-α	**59** (35.2-82.7)	**46.6** (20.1-94.1)	**52.6** (20.6-97.1)	1.1	0.9
TNF-β	**87.3** (39.3-131.1)	**93** (13.5-170.5)	**179** (10-773.2)	0.5	0.5

Table shows median concentrations in bold and interquartile range (IQR).

**Table 4B:** Saliva cytokine concentrations from Behçet’s Disease (BD) patients taking colchicine (COLC) and/or Azathioprine (AZA).

Cytokines	COLC	AZA	COLC+AZA	Neither
pg/ml	N=5	N=4	N=4	N=7
IL-1β	**1466** (755.2-3030)	**1340** (998.4-2053)	**1421** (752.4-2057)	**1667** (546.5-3703)
IL-2	**173.6** (156-260.6)	**176.9** (81.2-232.4)	**135.1** (66.6-317.9)	**179.3** (166.4-276.9)
IL-4	**89.6 **(78.7-112.9)	**77.6** (13-113.3)	**87.7** (19.7-179.7)	**93.1** (47-100)
IL-5	**52.8** (51.8-203)	**86** (62.8-110)	**73** (12.7-129.4)	**74.8** (40.9-134)
IL-6	**0.01** (0.01-82.1)	**18.4** (2.6-86.6)	**13.3** (2.9-40.2)	**23.7** (2.3-136.6)
IL-8	**494.8** (310.6-2089)	**544.7** (378.1-2009)	**835.6** (333.6-910)	**572.8** (325.4-1953)
IL-10	**33.6** (25-386.3)	**127.6** (39.31-156.2)	**70.8** (42.2-122.6)	**56.9** (22.4-162.6)
IL-12p70	**40.8** (0.01-506.5)	**157.7** (54.4-180.6)	**49.9** (2.8-108.2)	**82** (39.5-241.5)
IL-17A	**31.8** (16.9-72.6)	**36.1** (6-57.7)	**9.9** (0.5-59.7)	**27.2** (19.4-57.6)
IFN-γ	**0.01** (0.01-138.8)	**0.01** (0.01-80.1)	**0.01** (0.01-149.6)	**22** (0.01-93.5)
TNF-α	**63.4** (33.3-122.8)	**51.3** (18.7-94.7)	**46.6** (21.1-75.9)	**52.6** (28.3-97.1)
TNF-β	**53.9** (26.1-321.5)	**101.1** (62.3-157.6)	**66** (0.01-157.7)	**98.5** (38.3-179)

Bold the median values in the 3 columns and interquartile range (IQR) in BD patients identified as taking COLC or AZA, both or neither AZA or COLC. COLC refers only to the exclusion of AZA and vice-versa, however this does not exclude other medications that the patient may have been taking.

### Normalised Salivary Cytokine Levels

**Figure 3:**
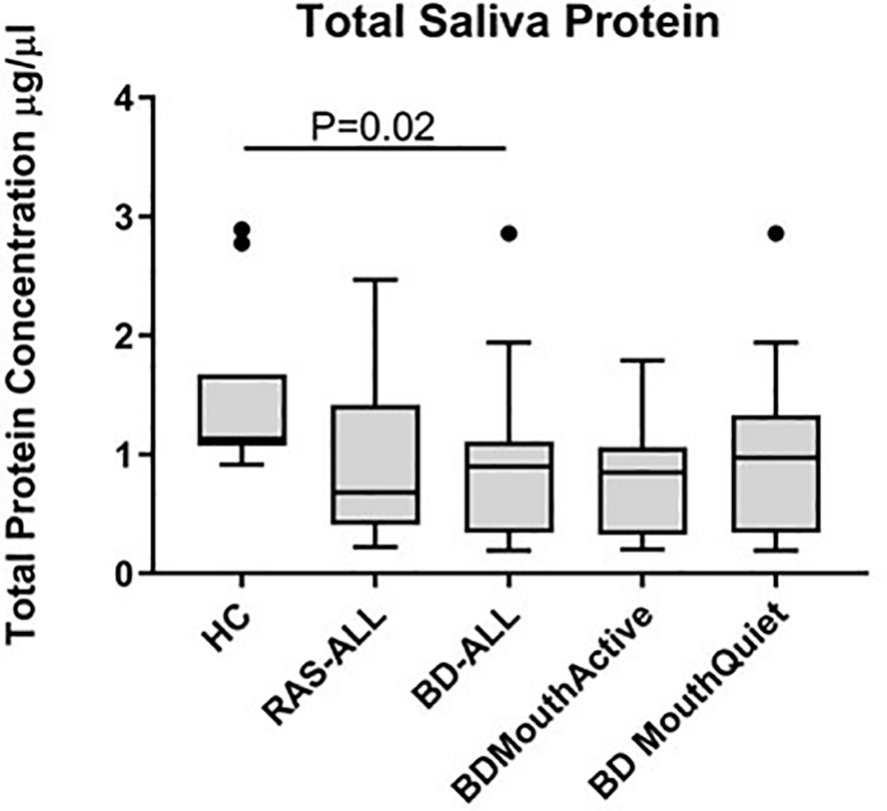
Total protein concentrations in saliva of healthy controls (HC), Behçet’s Disease (BD) and recurrent aphthous stomatitis (RAS) patients. The Tukey box plots show levels in BD-ALL (N=20), RAS-ALL (N=7) and HC (N=10). The BD patients were further grouped into patients with oral ulceration, BD Mouth Active (N=9), and patients with no oral ulceration, BD Mouth Quiet (N=11). Mann-Whitney U test, significance based on two tailed 95% confidence interval (CI). Exact P values are indicated.

**Figure 4:**
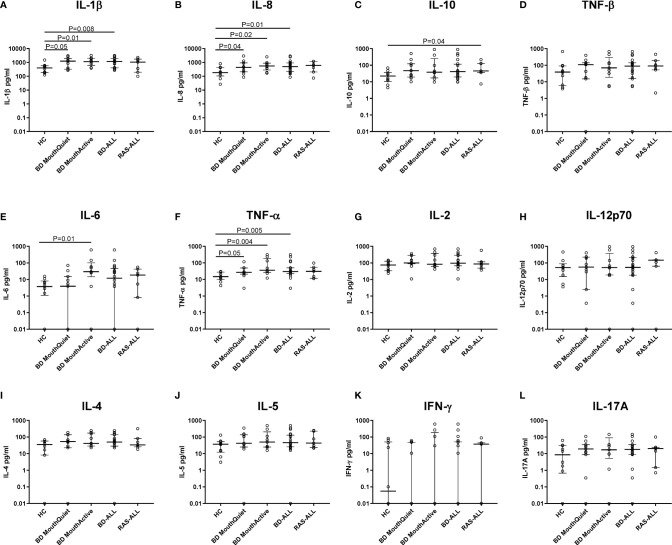
Saliva cytokine levels normalised to total protein differentially expressed in BD and RAS patients as compared to HCs. Plots show the cytokine concentrations (pg/ml) from BD-ALL (N=20), RAS-ALL (N=7) and HC (N=10). The BD patients were further grouped into patients with oral ulceration, BD Mouth Active (N=9), and patients with no oral ulceration, BD Mouth Quiet (N=11). **(A)** IL-1β, **(B)** IL-8, **(C)** IL-10, **(D)** TNF-β, **(E)** IL-6, **(F)** TNF-α, **(G)** IL-2, **(H)** IL-12p70, **(I)** IL-4, **(J)** IL-5, **(K)** IFN-γ, **(L)** IL-17A. The median and interquartile range are shown (Median ± IQR). Mann-Whitney U test, significance based on two tailed 95% confidence interval (CI). Exact P values are indicated.

**Figure 5:**
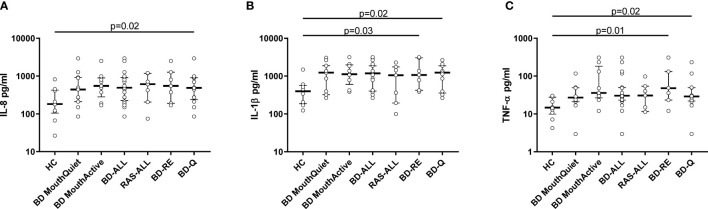
Disease Activity. Normalised cytokine levels differentially expressed in saliva from BD and RAS patients as compared to HC. Graphs show the concentrations of IL-8 **(A)**, IL-1β **(B)** and TNF-α **(C)** expressed in pg/ml from BD-ALL (N=20), RAS-ALL (N=7) and HC (N=10). The BD patients were further grouped into disease activity relapsed patients BD-RE (N=7) and disease activity quiet patients BD-Q (N=13). Data is presented as median ± IQR. Significant differences were determined by two tailed Mann-Whitney U test with 95% confidence interval (CI). Exact p values are indicated.

Total protein concentrations were measured in all saliva samples from BD, RAS and HCs and found to be significantly lower in BD patients than in HCs (p=0.02) (**Figure 3**). This prompted us to review the measured cytokine levels and normalise them to the total protein concentration. Following normalisation of the data, IL-1β, IL-6, IL-8, IL-10 and TNF-α concentrations were all significantly higher in BD than in HCs with only IL-10 concentrations significantly higher in RAS than in HCs (p=0.04) (**Figure 4** and **Table 5**). To determine if normalised cytokine saliva levels differed with disease activity, we investigated the levels of all the cytokines in BD-RE and BD-Q patients and found that IL-1β and TNF-α concentrations were significantly higher in both BD-RE and BD-Q when compared to HC saliva (**Figure 5**). IL-8 was also significantly higher in BD-Q than HCs (p=0.02). Further analysis of the treatment regimen with the normalised saliva data revealed that salivary concentrations of IL-10 (p=0.03) and IL-12p70 (p=0.03) were significantly lower in patients taking both COLC and AZA than BD patients treated with only AZA. IL-1β concentrations were also lower in BD patients taking both medications, however these levels did not reach significance (p=0.06) (**Supplementary Figure S3**).

**Table 5:** Concentrations of cytokines in normalized saliva of BD patients presenting with oral ulceration, Mouth Active (BD-MA), or without oral ulceration, Mouth Quiet (BD-MQ) compared to HC.

Cytokines	Group	Normalized Saliva		p-values*
		Median	IQR	
IL-1β	HC	399.3	187.5, 571	
	BD-MQ	1239	324.8, 1887	**0.05**
	BD-MA	1126	605.8, 1978	**0.01**
	BD-ALL	1182	403.4, 1883	**0.008**
IL-2	HC	74.3	34, 126	
	BD-MQ	99.1	90.4, 283.4	0.11
	BD-MA	84.0	62.1, 372	0.18
	BD-ALL	96.6	67.2, 289.4	0.08
IL-4	HC	35.2	8.1, 57.4	
	BD-MQ	53.6	22.4, 136.1	0.31
	BD-MA	41.4	28.7, 173.2	0.13
	BD-ALL	50.6	28.3, 138.1	0.14
IL-5	HC	36.7	12.2, 52.3	
	BD-MQ	41.8	25.1, 136	0.25
	BD-MA	50.1	25, 205	0.25
	BD-ALL	46	25.5, 130	0.17
IL-6	HC	3.7	1.1, 8.1	
	BD-MQ	3.9	0.01, 15.1	0.92
	BD-MA	29.6	14.7, 101.1	**0.01**
	BD-ALL	12.1	0.01, 46.5	0.20
IL-8	HC	182.3	104.8, 426	
	BD-MQ	442.5	210.4, 925.6	**0.04**
	BD-MA	551.1	282.6, 893.2	**0.02**
	BD-ALL	493	226.2, 916.9	**0.01**
IL-10	HC	22.5	10.5, 36.8	
	BD-MQ	46.6	19.6, 122.2	0.10
	BD-MA	38	17.3, 253.2	0.11
	BD-ALL	41	19.6, 116.3	0.06
IL-12p70	HC	52.3	15.2, 89.3	
	BD-MQ	55.7	2.5, 223.8	0.92
	BD-MA	52.3	18.8, 374.2	0.80
	BD-ALL	54.0	18.2, 215.9	0.80
IL-17A	HC	8.5	0.7, 31.1	
	BD-MQ	19.0	8.8, 35.1	0.43
	BD-MA	17.0	5.2, 89.9	0.40
	BD-ALL	18.0	8.9, 35.0	0.32
IFN-γ	HC	0.06	0.01, 50.3	
	BD-MQ	0.01	0.01, 48.4	0.58
	BD-MA	0.01	0.01, 188.7	0.70
	BD-ALL	0.01	0.01, 50.7	0.90
TNF-α	HC	14.7	9.9, 27.8	
	BD-MQ	27.1	21.1, 49.3	**0.05**
	BD-MA	36.1	26.6, 182.8	**0.004**
	BD-ALL	30.4	23.2, 50.0	**0.005**
TNF-β	HC	39.3	6.0, 91.7	
	BD-MQ	109.0	15.1, 141.4	0.46
	BD-MA	69.5	19.0, 295.4	0.32
	BD-ALL	89.2	15.9, 141.2	0.30

*p-value when compared with HC saliva. Significant values are in bold.

Normalized saliva HC (N=10), BD-MQ (N=11), BD-MA (N=9). Median concentrations with interquartile ranges (IQR) are shown. Cytokines below the lower levels limits of detection were considered not detectable and therefore arbitrarily assigned concentrations of 0.01 pg/ml.

### Correlations Between Normalised Salivary Cytokine Levels Across BD Patients

**Figure 6:**
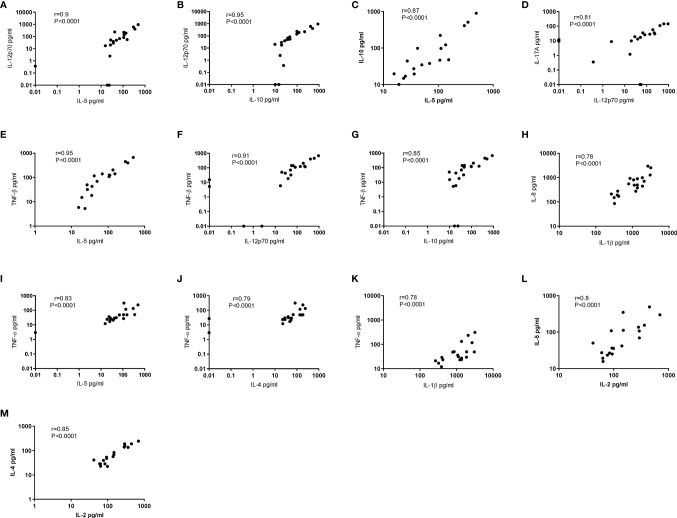
Correlation coefficients between 12 cytokines in Saliva. Correlation of normalised salivary cytokine concentrations in BD saliva (N=20). Spearman correlation was determined, r and p values with 95% CI. **(A)** IL-12p70 vs IL-5, **(B)** IL-12p70 vs IL-10, **(C)** IL-10 vs IL-5, **(D)** IL-17A vs IL-12p70, **(E)** TNF-β vs IL-5, **(F)** TNF-β vs vs IL-12p70, **(G)** TNF-β vs IL-10, **(H)** IL-8 vs IL-1β, **(I)** TNF-α vs IL-5, **(J)** TNF-α vs IL-4, **(K)** TNF-α vs IL-1β, **(L)** IL-5 vs IL-2, **(M)** IL-4 vs IL-2.

To determine if there was a relationship between the different cytokines in saliva, we used Spearman’s correlation analysis. Correlation analyses between the different cytokines and normalised BD saliva samples showed that IL-12p70, IL-10, TNF-β and TNF-α concentrations positively correlated with IL-5 (p<0.0001) (**Figures 6A, C, E, I**). IL-12p70 and TNF-β also highly correlated with each other r=0.9 (p<0.0001) (**Figure 6F**) IL-12p70 and IL-10 (r=0.95, P<0.0001) (**Figure 6B**). Additional correlations were found between other BD salivary cytokines and these are summarised in **Table 6**.

**Table 6:** Correlation matrix of 12 cytokines in normalised saliva.

	IL-1β	IL-2	IL-4	IL-5	IL-6	IL-8	IL-10	IL-12p70	IL-17A	IFN-γ	TNF-α
IL-1β											
IL-2	0.54135										
IL-4	0.43400	**0.8522**									
IL-5	0.61654	**0.8015**	0.74539								
IL-6	0.16382	0.37641	0.19781	0.18059							
IL-8	**0.77744**	0.38346	0.35276	0.51278	0.01753						
IL-10	0.41654	0.63759	0.55961	**0.87068**	0.08382	0.25564					
IL-12p70	0.44152	0.71606	0.64409	**0.90034**	0.07203	0.29109	**0.94998**				
IL-17A	0.28808	0.6273	0.54251	0.68447	0.03087	0.26326	0.7296	**0.80963**			
IFN-γ	0.08486	0.39462	0.38967	0.46845	0.08428	-0.0178	0.52107	0.58154	0.58069		
TNF-α	**0.77594**	0.73083	**0.79353**	**0.83308**	0.0061	0.73684	0.65263	0.70177	0.59195	0.37425	
TNF-β	0.60098	0.77849	0.72573	**0.95374**	0.17303	0.56337	**0.84919**	**0.90971**	0.73288	0.42533	0.82287

Correlation Matrix of BD normalised saliva concentrations. Spearman’s correlation was calculated and r values are shown; those plotted in **Figure 6** are in bold.

## Discussion

This pilot study investigated the use of FlowCytomix™ multiplex assays to measure multiple cytokines in matched serum and saliva samples to explore their differential expression and potential as diagnostic or activity markers for BD. Recurrent ulceration is a primary symptom of BD and precedes systemic systems implying that oral ulceration is a key event in this disease. Numerous studies have reported differences in systemic cytokines in BD serum, but only a handful of studies investigating inflammatory markers in BD saliva have been carried out (68–71) including our own recent work (38). Saliva is a highly dynamic fluid that has the potential to reveal the on-going pathology of BD. The potential of saliva as a diagnostic tool for oral and systemic disease has been an exciting development in clinical medicine in recent years (72–75).

Cytokines have the capacity to act in an autocrine, paracrine and endocrine manner so that their effects can be both local and distal with considerable variations in biodynamic range (76–79), (**Supplementary Figure S4**). Our study suggests that the range of cytokines in serum and saliva are very different with Th1, Th2 and IL-17A cytokines more abundant in saliva than in serum across all groups. After normalizing the protein content, saliva samples provided valuable information on the state of oral immunity in all groups as well as key inflammatory markers in BD patients during dormant oral symptoms.

ELISA has been the “Gold Standard” cytokine detection method with commercial assays varying in detection limits. A BD study conducted by Zouboulis, et al., reported that up to 51% of their serum samples were below detectable limits of their ELISA assay (10 ng/ml) (80). Using the FlowCytomix™ array, we had an improved yield with 100% of BD saliva samples detected above the lower limit and only two out of the 36 serum samples below detection. Other analyte targets in serum were not as readily detected. Excluding IL-8 and TNF-α (**Table 2**), the majority of undiluted serum samples were below detectable limits. Using the same small volume (25 µl) with saliva revealed better cytokine detection than serum. Saliva had an average of only 3.3 out of 37 saliva samples (9%) per analyte below the assay detectable levels, the exception being salivary IFN-γ which had 21 saliva samples below the 1.6 pg/ml assay detection limit (**Table 2**).

### IL-8 and IL-1β

IL-1β and IL-8 concentrations were elevated in BD and RAS saliva compared to HCs, however, in serum, IL-1β levels were very low, and IL-8 concentrations were higher only in BD patient serum. IL-8 has been one of the main serum chemokines studied in BD and has been detected in significantly high concentrations compared to HC but not consistently between active and inactive episodes of disease (81). BD patients with active vascular clinical presentation had a 4-5-fold increase of IL-8 over those without any vascular symptoms, while IL-8 concentrations in inactive BD patients with a history of vasculitis were still 2-fold higher than inactive BD with no vascular association (82). An increase in IL-8 mRNA has been shown to be directly associated with BD serum-treated macrophages from HC *in vitro* (83) and although there was a diverse source of IL-8 from various cells (including neutrophils, monocytes, macrophages, endothelial and epithelial cells), lymphocytes were considered to be the major contributors (84).

IL-8 is a potent chemokine eliciting a strong immune response to a variety of stimuli, recruiting neutrophils, activating leukocytes in response to bacterial antigens and enhancing adherence of circulating leukocytes to endothelial cells during inflammation (81). Neutrophils and lymphocytes express the IL-8 receptor (85) as do monocytes and NK cells (86). Interestingly IL-8 was increased in RAS saliva but not serum, suggesting that while the ulcers may have similar morphologies to those of BD, RAS remains a local condition without the systemic manifestations associated with BD.

IL-1β is recognised as a “master cytokine” in inflammation, inducing other cytokines and acting as the main mediator coordinating attacks on invading microbes (87, 88) or responses to injury. Produced mainly by blood monocytes, but also from macrophages, dendritic cells, and neutrophils, it increases fever and hypotension. Increased levels of IL-1β has been shown to simultaneous increase in IL-8. IL-1β has been investigated in BD, but not to the extent of IL-8 or TNF-α. A significant rise of serum IL-1β has been detected in BD active and inactive patients compared to HC (89). Importantly, IL-8 has also been suggested as an inducer of matrix metalloproteinase which may have a role in ulcer development (90).

During oral ulceration, higher levels of IL-8 and IL-1β reflect mucosal damage and recruitment of inflammatory cells into the wound. Interestingly, in this study both BD-MQ and RAS-MQ (RAS data not included) had higher levels of salivary IL-8 and IL-1β to that of HCs, suggesting their continued presence during non-ulcer periods may reflect the mucosal instability of these patient groups. Furthermore, salivary IL-1β tended to be higher in quiet, systemically inactive, BD-Q patients than in relapsed patients. We have recently described high levels of neutrophil elastase in BD saliva and have proposed a working model of BD oral environment which involves neutrophil recruitment and IL-8 and IL-1β secretion (38).

In ELISA based assays comparing sera from patients with or without oral ulceration, IL-8 was significantly increased in individuals with oral ulcers (80, 82, 91). Importantly, our study also found that IL-8 concentrations were higher in serum of orally active BD patients compared to HCs and confirms that these levels are also high in orally active saliva, thereby indicating that the local high IL-8 mucosal response is mirrored in systemically higher IL-8 levels in the serum of orally active patients.

IL-8 is also produced by gingival epithelial cells in response to oral microbiota *in vitro* (92) and by nicotine (93), while a recent review has referenced data showing upregulation of IL-8 in oral diseases such as Oral Lichen Planus and periimplantitis with contradictory results in some studies of oral Leukoplakia (75). IL-8 can exist as a monomer, dimer or as a mixture of both and has profound effects on chemokine receptors CXCR1 and CXCR2. These molecules are important in neutrophil recruitment and activation and the monomeric form of IL-8 is thought to bind to glycosaminoglycans, which are present in the oral mucosal pellicle (94, 95).

The ratio of salivary IL-8 and IL-1β (average ratio of 1:2) showed a strong predictability that IL-1β would be nearly twice as high than IL-8 in 89% of the samples. Together, they showed a correlation in the collective saliva samples from all groups. However, serum samples did not adhere to this ratio. Furthermore, in normalised BD saliva samples, IL-1β and IL-8 showed a strong correlation (r=0.78, p<0.0001). While IL-8 has been suggested as a serological marker for assessing BD activity (91), our data would suggest that IL-8 alone is not specific enough since both RAS and BD patients had high levels of this chemokine. However, IL-8 paired with IL-1β measurements in saliva, could improve the validity of pre-empting an active ulcer episode. The IL-8 and IL-1β correlation demonstrates the reliability of saliva samples to provide accurate data for the potential monitoring of inflammatory markers.

### IFNγ

IFN-γ was originally recognised as the main constituent of the Th1 response (96, 97). It is produced by cells of both the innate and acquired immune systems, namely NK cells and T-cells. IFN-γ targets B cells, macrophages, and endothelial cells activating the expression of Class II MHC molecules on the surface of these antigen presenting cells (98). Although BD is often recognised as a Th1 dominant response (44), there are studies that have conflicted with the classic generation of increased IFN-γ. For instance, IFN-γ was measured in serum of BD active patients and was found to be lower than BD quiet, RAS, and HC (99). In our study, only 16 out of 37 saliva samples and one out of 36 serum samples had detectable IFN-γ levels. The trend was for the IFN-γ IQR (interquartile range) to be higher in the saliva from BD patients with active ulcers, which illustrated that only when the oral mucosa is damaged does the classic IFN-γ drive forward the Th1 response. Frassanito, et al, also found increased circulating IFN-γ from only symptomatic BD patients when compared to BD quiet and HCs (44).

### TNF-α

TNF-α is a rapidly released cytokine that can act as an alarm against foreign or stress stimuli (100). Mainly produced by monocytes and macrophages it can also be released from T-cells, neutrophils, mast cells, endothelial cells and keratinocytes (101–103). In this study, saliva TNF-α was significantly increased in both BD patients with and without oral ulcers. For those with oral ulcers, TNF-α likely plays a beneficial role in recruiting immune cells to battle pathogens associated with the wound. However, an increased presence of TNF-α in the mouth of BD patients without any ulcers could lead to an influx of neutrophils which as we have previously proposed can lead to mucosal instability following an exacerbated inflammatory cycle (38).

Although RAS-MA patients had the highest TNF-α median concentration in saliva (data not shown), due to the small sample size comparisons were not able to be elucidated. More samples are required from the RAS patient group for a more accurate assessment since it has been shown to be highly expressed in RAS oral lesions (61). Interestingly, the highest median TNF-α levels were measured in BD-MQ and not BD-MA serum. This suggests that TNF-α may be involved in stimulating a systemic response in BD. TNF-α has been one of the most intensively investigated cytokines in BD (41). BD patients with active uveitis have been shown to have significantly higher levels of TNF-α than those without any eye involvement for 3 months (104) and anti-TNF-α therapy (such as Infliximab) has been helpful in controlling active episodes of uveitis in BD (105) as well as oral and genital ulceration (106). For RAS patients, an *in vitro* study has shown that TNF-α was higher in unstimulated peripheral blood monocytes from RAS patients with ulcers than in HCs (107). Collectively, the data indicates that TNF-α has an important role in BD and RAS pathologies.

### IL-2 and IL-12p70

IL-12p70, another pro-inflammatory cytokine which is known to drive the Th1 immune response by inducing IFN-γ and IL-2 (108), was found to be increased in RAS saliva. Although this did not lead to higher levels of IL-2, IFN-γ was higher in RAS saliva (IQR 0.01-113.7pg/ml) than BD and HCs. In all BD patients, normalised IL-12p70 also showed a strong correlation with TNF-β in saliva (r=0.9, p<0.0001), suggesting their simultaneous presence is worthy of further investigation for potential synergistic activity. In *ex-vivo* studies of inflamed gut mucosa IL-12 and TNF-α could be induced by stimulating the cells with Human HSP-70 (109) a molecule that has been implicated in BD pathogenesis.

Elevated IL-2 levels have been reported in sera from BD patients with uveitis compared with non-uveitis patients (110) and in a mixed BD cohort with active disease but where only 6/44 patients had uveitis compared with healthy controls (111). In our study, serum samples from RAS-MA (n=2) had higher levels of IL-2 compared to RAS-MQ patients, however, since the sample size is small this warrants further investigation. Similarly, high median levels were also observed in BD-MA serum possibly reflecting the ongoing inflammatory process during oral ulceration. In saliva, however, similar IL-2 levels were detected in all groups. This indicates that IL-2 detected in saliva was not necessarily affected by oral ulceration in our cohort. IL-2 can also be influenced by IL-6. IL-6 can promote T helper and cytotoxic T-cell proliferation by increasing their IL-2 secretion (112, 113).

### IL-6 and IL-17

IL-6 is produced by T-cells, B cells, and macrophages and can stimulate the proliferation of B cells and immunoglobulins as well as differentiate T lymphocytes (114, 115). Naïve T-cells can be stimulated by IL-6 and transforming growth factor beta to become IL-17A secreting Th17-type cells (116). An increased level of IL-17A has been detected in BD serum (117) and the IL-23/IL-17 pathway is seen as crucial in several autoimmune diseases such as rheumatoid arthritis that had previously been regarded at Th1 related disease (118, 119). In addition to CD4+ Th17 cells, IL-17A is produced by γδ T cells, NK cells (120), and is secreted by CD3+ (CD4-/CD8-) cells infiltrating salivary glands in Sjögrens syndrome (121) and in the plasma from those with systemic lupus erythematosus (122). In response to extracellular pathogens, IL-17 can induce the production of IL-8 thus recruiting neutrophils and contributing to inflammation. However when the release of IL-17A is exacerbated, this can also lead to the destructive tissue pathologies of inflammatory and autoimmune diseases (123). IL-17A was incorporated into our multiplex assay panel because it has been shown to recruit neutrophils whose proteases can cause detrimental damage to the mucosa (124). IL-17A has not previously been measured in saliva of BD or RAS patients, but has been investigated in chronic periodontitis in which it was significantly lower than HC (125). There is conflicting ELISA data in studies of IL-17 in BD. Chi, et al., failed to detect IL-17A in BD sera (117), while Shen, et al., reported serum IL-17 levels were significantly higher in BD serum compared to HCs and in BD patients with uveitis than those without (126). Saedeghi, et al., measured serum IL-17 levels in BD but found no significant differences between patients with and without uveitis (110). We also found IL-17 difficult to detect in serum (18.9% of samples above lower limits of detection over all groups). However, in saliva 89.2% of samples were above the lower limits of detection. This may reflect the sensitivity of the assay methods as Gholijani, et al., using a multiplex cytokine assay system, reported similar IL-17 levels to our study, however, they found significant differences between BD and healthy control sera, possibly reflecting the larger sample size (n=44 for BD and HC) and recent diagnosis (111).

Serum IL-6 was detected in only five samples across the cohort. In contrast, salivary IL-6 was significantly higher in BD-MA compared to HCs and BD-MA and BD-MQ although, normalised IL-6 salivary levels were significantly higher only in BD-MA, again, this was not reflected in IL-17A saliva levels. Low serum IL-6 in BD patients but high individual IL-17A measurements could be due to IL-17A production by a non-classical Th17 type cell such as γδ T cells or NK cells (120) which does not depend on IL-6 stimulation. However, these cytokine measurements require a larger samples size to confirm that IL-17A is independent of IL-6 in this system. IL-6 has previously been undetectable in BD and HC plasma using ELISA with detection limits of 18 pg/ml (114). The IL-6 detection limit for our FlowCytomix array was 1.2 pg/ml, and although evidence of the presence of IL-6 was measured in serum, our data coincides with that of other previous reports which state that serum IL-6 was absent or at particularly low levels in BD and HC. However, in another study IL-6 was significantly increased in BD patients compared to RAS and HC Using a similar multiplex bead assay to our own, many of their samples were below the level of detection. Yet, IL-6 was still described by Curnow, et al, as one of the most abundant inflammatory cytokines amongst a panel of 10 detected in serum (127). In our study, the majority of serum samples had no detectable IL-6; however, it was detected in most saliva samples, showing the highest levels in the BD group with active ulcers. Since a decreased production of IL-6 in the oral mucosa is thought to drive rapid oral mucosal healing (128) its presence in BD-MA could perpetuate the ulcer. The results of our data do not support the previous hypothesis that IL-6 has a prominent role to play in systemic inflammation in BD but it does suggest that oral mucosa inflammatory cells release IL-6 in response to oral ulceration. Early work on the influence of IL-6 in periodontitis (129) suggested it might be protective. It has also been suggested that mastication can up regulate Th17 cells in inflamed gingival mucosa (130).

### IL-4 and IL-5

IL-4 and IL-5 are Th2-type cytokines that stimulate B cells to proliferate and mature. IL-5 also promotes maturation of eosinophils. IL-4 is considered a critical element for driving the development of Th2 immune response (131). In our study, IL-4 levels were detectable in only a few serum samples (mainly BD-MQ) but in almost all saliva samples. However, while RAS levels were lower than BD and HCs there was no significant differences between patient groups and HCs, therefore, IL-4 may be a more prominent force driving the production of antibodies in the mouth other than systemically driving a Th2 response. This is possibly a strategy for maintaining levels of antibodies such as SIgA which has important heterotypic functions, such as viral agglutination, over and above antigenic specificity, in oral mucosal tissue that is constantly turning over and where fluids from salivary gland and gingival crevicular fluid are constantly replenished, compared to the closed circulatory system.

### IL-10

IL-10 is also a key immunoregulatory cytokine produced by almost all leukocytes, and Th17 type cells and has previously been found to promote would healing (132, 133). IL-10 has been found in high levels in active BD patient serum (99) but has not significantly increased in highly active BD compared to inactive or mild disease (134). IL-5 and IL-10 showed a strong correlation in normalised BD saliva samples. Both of these Th2-type cytokines can promote B cell proliferation and maturation leading towards a Th2 response. While IL-10 can help to regulate the Th1/Th2 balance it also inhibits T-cells from making IFN-γ which would explain the low levels in our cohort (135).

### Medication

The effect of medication on salivary cytokines was difficult to unravel with few significant differences between the treatments and the small sample size, however, it was noted that the combination of COLC+AZA showed lower median levels of both a pro-inflammatory cytokine (IL-12p70) and an anti-inflammatory cytokine (IL-10) to a significant extent. Normalised IL-1β salivary levels were also lower with the combination treatment however, failed to reach significance. It is rare to find an individual with BD or who is being investigated for BD that is treatment naïve. Therefore, it is difficult to assess a baseline cytokine profile not influenced by immunoregulatory medications. For this pilot study, the findings are a first look at the influence of pro-inflammatory cytokines in saliva in this cohort.

### Saliva as a Biomarker for Diagnosis and Disease Progression

Other systemic diseases in which oral lesions arise such as systemic lupus erythematosus (SLE) and oral cancer have also had their saliva cytokines investigated. In Marques, et al’s study, they found an increase in IFN-γ, IL-10, IL-17, IL-1β, IL-6, and IL-4 in SLE patients without periodontal disease compared to HC (136). Also in a meta-analysis, IL-8, IL-1β, TNF-α, IL-6, and IL-1α were found to be increased in oral cancer compared to HC and leukoplakia and were suggested as cancer biomarkers (137) In our study, the significant differences between HC and BD-MQ is an important finding to be able to differentiate the levels of cytokines present in “normal” saliva content and orally asymptomatic BD patients saliva. Increased detection of IL-8, IL-1β, and TNF-α may not be specific to BD for diagnostics, but can still be useful to monitor the level of oral inflammation in order to deter further ulcerations, and potentially, accompanying systemic manifestations.

Homeostatic regulation in the oral cavity and systemically involves a pattern of cytokine expression which may appear to be nonspecific. However, recent findings by ter Horst et al. and Li et al. suggest a complex pattern which is more individual than expected (138, 139). Ter Horst et al. (2021) also suggest a seasonality in some responses (140), and anecdotally this has been observed in BD patients with worsening symptoms during autumn. Age related changes have also been cited by these studies and IL-6 associated quantitative trait loci were identified. Key cytokines in their studies were IL-18 and IL-22 which were not included in our panel. However, it is our hypothesis that that the balance of various cytokines in HC, RAS, BD-MA and BD-MQ might be quite different and provide a panel that might aid differential diagnosis of this difficult disease. Inclusion of IL-15, IL-22 and IL-18, or indeed other cytokines, in future multiplex studies might be more revealing in future expanded studies.

Salivary cytokine concentrations were normalised to total protein after we observed that the volume collected differed between patients over the same collection time and some patients found it more difficult to produce saliva than others. The difficulty that some patients may have in producing saliva may be related to their medication regime and may not be as a direct effect of BD. Indeed, we found that the total protein concentration varied between patients and that in BD this was significantly lower than in HC. Furthermore, in the mouth there is a constant turnover of saliva that is lost through swallowing, whereas in serum the protein concentration was assumed to be fairly constant with clinical reference values at between 60-80g/L (141).

It is worth bearing in mind that serum and plasma samples are derived from a closed system while saliva is the pool of materials directly secreted by the salivary glands but with the potential for contributions from serum exudates from the gingival margins or indeed secretions from cells within the mucosal layers. The oral tissues are also covered in a “Mucosal Pellicle” which is known to be an active environment (95, 142) where heterotypic associations between signalling molecules and receptors has the potential for enhancing signalling cascades.

It should also be noted that the surface epithelium of the oral mucosa is in a constant state of loss and regeneration and the stability/integrity of the mucosa is dependent on a strict homeostasis. With that in mind we are cognisant that the fluid in the oral cavity, “saliva”, is a highly dynamic fluid that has the potential to reveal the ongoing pathology of this disease. The potential of saliva as a diagnostic tool for oral and systemic disease has been an exciting development in clinical medicine in recent years (72–75).

We are not able to say whether there are receptors in the mouth for all the cytokines we measured as well as what the half-life of these cytokines and chemokines is in saliva and how sensitive these cytokines are to the dynamic pH changes in saliva. Nor, indeed if they are in monomeric, dimeric or in heterotypic associations which may make their detection more difficult. However, as more studies of oral diseases and other conditions are carried out using saliva, we feel that our approach to investigating a panel of cytokines that might define or predict disease progression using a non-invasive sample is of significant value in support of patients with BD and other inflammatory diseases (143).

### Limitations of Study

BD is a rare disease and therefore presents challenges when designing an investigative study. This pilot study consisted of 20 BD patients, 7 RAS patients, and 10 HC. Of this small cohort, only 15% of the BD patients were treatment naïve at the time of sampling. Ideally, all samples would be from untreated patients, however we have tried to limit the cohort to drugs with similar actions and have excluded patients who were taking biologics. For future studies, newly diagnosed patients who have not started medication should also be compared with those on established anti-inflammatory medications. Also, a power calculation should be carried out to establish the minimum number of patients and controls for a more robust statistical analysis. A larger RAS cohort, with an equal number of individuals with and without ulcers, would also provide meaningful disease control data especially when analysing saliva samples. The following criteria should be taken into account for BD and RAS patients: ulcer size, duration, and number present at the time of sampling, years since diagnosis/symptoms, HLA-B51 status and/or geographic origins. For all patients and HCs: smoker status, alcohol consumption, periodontal/caries status and oral hygiene habits.

### Summary

Oral ulceration is the most common characteristic of BD and is often predictive of the onset of systemically active disease. This pilot study used FlowCytomix™ multiplex assays to measure multiple cytokines in matched serum and saliva samples to explore their differential expression and potential as diagnostic or activity markers for BD.

The elevated saliva cytokines IL-8, IL-1β, and TNF-α in BD patients without oral ulcers could be included in a potential cytokine panel to assist in the differential diagnosis, predicting the reoccurrence of ulcers or systemic relapse, and monitoring treatments. There are clear benefits for using saliva over serum for the detection of cytokines and multiplex assays lend themselves to screening with limited sample volumes. Normalisation of saliva samples to total protein was found to be key in standardising this highly dynamic specimen.

Our study highlighted that IL-1β, IL-8, TNF-α, IL-6 and IL-10 saliva cytokines play a role perpetuating BD oral inflammation and should be incorporated in further investigations. IL-10 and IL-6 would also be of interest in dissecting the immune dysregulation in RAS. Although the IL-17A measurements were unexpectedly low, the IL-17/IL-23/IL-22/IL-6 axis might benefit from further elucidation in a larger cohort. In a disease such as BD where the spectrum of manifestations requires highly individualised and patient specific interventions, these methodologies are of considerable value to support frequent monitoring of the episodic disease and potentially predict and prevent relapse.

## Data Availability Statement

The raw data supporting the conclusions of this article will be made available by the authors, without undue reservation.

## Ethics Statement

The studies involving human participants were reviewed and approved by the City and East London (P/03/122). The patients/participants provided their written informed consent to participate in this study.

## Author Contributions

TN, MH, LB, and EH-P contributed to conception, design, data acquisition and analysis. LB, TN, and EH-P drafted and critically revised the manuscript. MH critically revised the manuscript. FF helped with the data analysis and interpretation as well as critically reviewed the manuscript. All authors gave their final approval and agreed to be accountable for all aspects of the work.

## Funding

Financial support provided by the James Paget PhD Studentship to TN.

## Conflict of Interest

The authors declare that the research was conducted in the absence of any commercial or financial relationships that could be construed as a potential conflict of interest.

## Publisher’s Note

All claims expressed in this article are solely those of the authors and do not necessarily represent those of their affiliated organizations, or those of the publisher, the editors and the reviewers. Any product that may be evaluated in this article, or claim that may be made by its manufacturer, is not guaranteed or endorsed by the publisher.

## References

[1] Marshall SE. Behçet's Disease. Best Pract Res Clin Rheumatol (2004) 18(3):291–311. doi: 10.1016/j.berh.2004.02.008 15158742

[2] Deuter CM Kötter I Wallace GR Murray PI Stübiger N Zierhut M. Behçet's Disease: Ocular Effects and Treatment. Prog Retin Eye Res (2008) 27(1):111–36. doi: 10.1016/j.preteyeres.2007.09.002 18035584

[3] Verity DH Wallace GR Vaughan RW Stanford MR. Behçet's Disease: From Hippocrates to the Third Millennium. Br J Ophthalmol (2003) 87(9):1175–83. doi: 10.1136/bjo.87.9.1175 PMC1771837 12928293

[4] Kural-Seyahi E Fresko I Seyahi N Ozyazgan Y Mat C Hamuryudan V. The Long-Term Mortality and Morbidity of Behçet Syndrome: A 2-Decade Outcome Survey of 387 Patients Followed at a Dedicated Center. Med (Baltimore) (2003) 82(1):60–76. doi: 10.1097/00005792-200301000-00006 12544711

[5] Abu-Ameerh MA Mohammed SF Mohammad MT Ababneh OH Al-Bdour MD. Ocular Manifestations of Behçet's Disease in Jordanian Patients. Saudi J Ophthalmol (2013) 27(4):247–51. doi: 10.1016/j.sjopt.2013.06.012 PMC3841248 24371419

[6] Alpsoy E. Behçet's Disease: A Comprehensive Review With a Focus on Epidemiology, Etiology and Clinical Features, and Management of Mucocutaneous Lesions. J Dermatol (2016) 43(6):620–32. doi: 10.1111/1346-8138.13381 27075942

[7] Greco A De Virgilio A Ralli M Ciofalo A Mancini P Attanasio G. Behçet's Disease: New Insights Into Pathophysiology, Clinical Features and Treatment Options. Autoimmun Rev (2018) 17(6):567–75. doi: 10.1016/j.autrev.2017.12.006 29631062

[8] Al-Otaibi LM Porter SR Poate TW. Behçet's Disease: A Review. J Dent Res (2005) 84(3):209–22. doi: 10.1177/154405910508400302 15723859

[9] Saadoun D Wechsler B. Behçet's Disease. Orphanet J Rare Dis (2012) 7:20. doi: 10.1186/1750-1172-7-20 22497990 PMC3472229

[10] Mendes D Correia M Barbedo M Vaio T Mota M Gonçalves O. Behçet's Disease–A Contemporary Review. J Autoimmun (2009) 32(3-4):178–88. doi: 10.1016/j.jaut.2009.02.011 19324519

[11] Criteria for Diagnosis of Behcet's Disease. International Study Group for Behcet's Disease. Lancet (London England) (1990) 335(8697):1078–80. doi: 10.1016/0140-6736(90)92643-V 1970380

[12] Davatchi F Assaad-Khalil S Calamia KT Crook JE Sadeghi-Abdollahi B Schirmer M. The International Criteria for Behçet's Disease (ICBD): A Collaborative Study of 27 Countries on the Sensitivity and Specificity of the New Criteria. J Eur Acad Dermatol Venereol (2013) 28:338–47. doi: 10.1111/jdv.12107 23441863

[13] Bernabe E Marcenes W Mather J Phillips C Fortune F. Impact of Behcet's Syndrome on Health-Related Quality of Life: Influence of the Type and Number of Symptoms. Rheumatology (2010) 49(11):2165–71. doi: 10.1093/rheumatology/keq251 20675710

[14] Mumcu G Ergun T Inanc N Fresko I Atalay T Hayran O. Oral Health Is Impaired in Behçet's Disease and Is Associated With Disease Severity. Rheumatol (Oxford) (2004) 43(8):1028–33. doi: 10.1093/rheumatology/keh236 15161982

[15] Direskeneli H. Behçet's Disease: Infectious Aetiology, New Autoantigens, and HLA-B51. Ann Rheum Dis (2001) 60(11):996–1002. doi: 10.1136/ard.60.11.996 11602462 PMC1753405

[16] Lehner T. Immunopathogenesis of Behcet's Disease. Annales Med Interne (1999) 150(6):483–7. 10615534

[17] Leccese P Alpsoy E. Behçet's Disease: An Overview of Etiopathogenesis. Front Immunol (2019) 10:1067. doi: 10.3389/fimmu.2019.01067 31134098 PMC6523006

[18] Direskeneli H. Autoimmunity vs Autoinflammation in Behcet's Disease: Do We Oversimplify a Complex Disorder? Rheumatol (Oxford) (2006) 45(12):1461–5. doi: 10.1093/rheumatology/kel329 16998234

[19] Yokota K Hayashi S Fujii N Yoshikawa K Kotake S Isogai E. Antibody Response to Oral Streptococci in Behçet's Disease. Microbiol Immunol (1992) 36(8):815–22. doi: 10.1111/j.1348-0421.1992.tb02083.x 1474932

[20] Lehner T Lavery E Smith R van der Zee R Mizushima Y Shinnick T. Association Between the 65-Kilodalton Heat Shock Protein, Streptococcus Sanguis, and the Corresponding Antibodies in Behçet's Syndrome. Infect Immun (1991) 59(4):1434–41. doi: 10.1128/iai.59.4.1434-1441.1991 PMC257860 2004821

[21] Lehner T. The Role of Heat Shock Protein, Microbial and Autoimmune Agents in the Aetiology of Behçet's Disease. Int Rev Immunol (1997) 14(1):21–32. doi: 10.3109/08830189709116842 9203024

[22] Lehner T. Immunological Aspects of Recurrent Oral Ulceration and Behçet's Syndrome. J Oral Pathol (1978) 7(6):424–30. doi: 10.1111/j.1600-0714.1978.tb01613.x 105103

[23] Seoudi N Bergmeier LA Hagi-Pavli E Bibby D Curtis MA Fortune F. The Role of TLR2 and 4 in Behcet's Disease Pathogenesis. Innate Immun (2013) 20:412–32. doi: 10.1177/1753425913498042 23940075

[24] Krause I Mader R Sulkes J Paul M Uziel Y Adawi M. Behçet's Disease in Israel: The Influence of Ethnic Origin on Disease Expression and Severity. J Rheumatol (2001) 28(5):1033–6. 11361184

[25] Pineton de Chambrun M Wechsler B Geri G Cacoub P Saadoun D. New Insights Into the Pathogenesis of Behcet's Disease. Autoimmun Rev (2012) 11(10):687–98. doi: 10.1016/j.autrev.2011.11.026 22197900

[26] Verity DH Marr JE Ohno S Wallace GR Stanford MR. Behcet's Disease, the Silk Road and HLA-B51: Historical and Geographical Perspectives. Tissue Antigens (1999) 54(3):213–20. doi: 10.1034/j.1399-0039.1999.540301.x 10519357

[27] Takeuchi M Kastner DL Remmers EF. The Immunogenetics of Behcet's Disease: A Comprehensive Review. J Autoimmun (2015) 64:137–48. doi: 10.1016/j.jaut.2015.08.013 PMC4628864 26347074

[28] Hasan A Fortune F Wilson A Warr K Shinnick T Mizushima Y. Role of Gamma Delta T Cells in Pathogenesis and Diagnosis of Behcet's Disease. Lancet (London England) (1996) 347(9004):789–94. doi: 10.1016/S0140-6736(96)90868-5 8622334

[29] Hasan MS Bergmeier LA Petrushkin H Fortune F. Gamma Delta (γδ) T Cells and Their Involvement in Behçet's Disease. J Immunol Res (2015) 2015:705831. doi: 10.1155/2015/705831 26539557 PMC4619955

[30] Hasan MS Ryan PL Bergmeier LA Fortune F. Circulating NK Cells and Their Subsets in Behcet's Disease. Clin Exp Immunol (2017) 188:311–22. doi: 10.1111/cei.12939 PMC5383445 28170096

[31] Mizuki N Meguro A Ota M Ohno S Shiota T Kawagoe T. Genome-Wide Association Studies Identify IL23R-IL12RB2 and IL10 as Behçet's Disease Susceptibility Loci. Nat Genet (2010) 42(8):703–6. doi: 10.1038/ng.624 20622879

[32] Remmers EF Cosan F Kirino Y Ombrello MJ Abaci N Satorius C. Genome-Wide Association Study Identifies Variants in the MHC Class I, IL10, and IL23R-IL12RB2 Regions Associated With Behcet's Disease. Nat Genet (2010) 42(8):698–702. doi: 10.1038/ng.625 20622878 PMC2923807

[33] Matsumura N Mizushima Y. Leucocyte Movement and Colchicine Treatment in Behcet's Disease. Lancet (London England) (1975) 2(7939):813. doi: 10.1016/S0140-6736(75)80031-6 78172

[34] Sakane T Takeno M Suzuki N Inaba G. Behçet's Disease. N Engl J Med (1999) 341(17):1284–91. doi: 10.1056/NEJM199910213411707 10528040

[35] Mege JL Dilsen N Sanguedolce V Gul A Bongrand P Roux H. Overproduction of Monocyte Derived Tumor Necrosis Factor Alpha, Interleukin (IL) 6, IL-8 and Increased Neutrophil Superoxide Generation in Behçet's Disease. A Comparative Study With Familial Mediterranean Fever and Healthy Subjects. J Rheumatol (1993) 20(9):1544–9. 8164212

[36] Yosipovitch G Shohat B Bshara J Wysenbeek A Weinberger A. Elevated Serum Interleukin 1 Receptors and Interleukin 1B in Patients With Behçet's Disease: Correlations With Disease Activity and Severity. Isr J Med Sci (1995) 31(6):345–8. 7607852

[37] Değer O Orem A Akyol N Bahadir S Yildirmis S. Polymorphonuclear Leukocyte Elastase Levels in Patients With Behçet's Disease. Clin Chim Acta (1995) 236(2):129–34. doi: 10.1016/0009-8981(95)06033-A 7554279

[38] Novak T Fortune F Bergmeier L Khan I Hagi-Pavli E. Neutrophil Elastase and Endogenous Inhibitors in Behçet's Disease Saliva. Clin Exp Immunol (2020) 202:93–105. doi: 10.1111/cei.13483 32580239 PMC7488119

[39] Williams DW Greenwell-Wild T Brenchley L Dutzan N Overmiller A Sawaya AP. Human Oral Mucosa Cell Atlas Reveals a Stromal-Neutrophil Axis Regulating Tissue Immunity. Cell (2021) 184(15):4090–104.e15. doi: 10.1016/j.cell.2021.05.013 34129837 PMC8359928

[40] Tong B Liu X Xiao J Su G. Immunopathogenesis of Behcet's Disease. Front Immunol (2019) 10:665. doi: 10.3389/fimmu.2019.00665 30984205 PMC6449449

[41] Zhou ZY Chen SL Shen N Lu Y. Cytokines and Behcet's Disease. Autoimmun Rev (2012) 11(10):699–704. doi: 10.1016/j.autrev.2011.12.005 22197901

[42] Ben Ahmed M Houman H Miled M Dellagi K Louzir H. Involvement of Chemokines and Th1 Cytokines in the Pathogenesis of Mucocutaneous Lesions of Behçet's Disease. Arthritis Rheum (2004) 50(7):2291–5. doi: 10.1002/art.20334 15248229

[43] Curnow SJ Pryce K Modi N Knight B Graham EM Stewart JE. Serum Cytokine Profiles in Behcet's Disease: Is There a Role for IL-15 in Pathogenesis? Immunol Lett (2008) 121(1):7–12. doi: 10.1016/j.imlet.2008.07.009 18706446

[44] Frassanito MA Dammacco R Cafforio P Dammacco F. Th1 Polarization of the Immune Response in Behçet's Disease: A Putative Pathogenetic Role of Interleukin-12. Arthritis Rheum (1999) 42(9):1967–74. doi: 10.1002/1529-0131(199909)42:9<1967::AID-ANR24>3.0.CO;2-Z 10513814

[45] Touzot M Cacoub P Bodaghi B Soumelis V Saadoun D. IFN-α Induces IL-10 Production and Tilt the Balance Between Th1 and Th17 in Behçet Disease. Autoimmun Rev (2015) 14(5):370–5. doi: 10.1016/j.autrev.2014.12.009 25541082

[46] Kim J Park JA Lee EY Lee YJ Song YW Lee EB. Imbalance of Th17 to Th1 Cells in Behçet's Disease. Clin Exp Rheumatol (2010) 28(4 Suppl 60):S16–9. 20868565

[47] Schett G Elewaut D McInnes IB Dayer JM Neurath MF. How Cytokine Networks Fuel Inflammation: Toward a Cytokine-Based Disease Taxonomy. Nat Med (2013) 19(7):822–4. doi: 10.1038/nm.3260 23836224

[48] Striz I Brabcova E Kolesar L Sekerkova A. Cytokine Networking of Innate Immunity Cells: A Potential Target of Therapy. Clin Sci (Lond) (2014) 126(9):593–612. doi: 10.1042/CS20130497 24450743

[49] Hamedi M Bergmeier LA Hagi-Pavli E Vartoukian SR Fortune F. Differential Expression of Suppressor of Cytokine Signalling Proteins in Behcet's Disease. Scandinavian J Immunol (2014) 80(5):369–76. doi: 10.1111/sji.12211 25207681

[50] Tulunay A Dozmorov MG Ture-Ozdemir F Yilmaz V Eksioglu-Demiralp E Alibaz-Oner F. Activation of the JAK/STAT Pathway in Behcet's Disease. Genes Immun (2015) 16(2):170–5. doi: 10.1038/gene.2014.64 PMC4443896 25410656

[51] Fortune F. Can You Catch Behçet's Disease? J Lab Clin Med (2003) 141(1):5–6. doi: 10.1067/mlc.2003.2 12518164

[52] Seoudi N Bergmeier LA Drobniewski F Paster B Fortune F. The Oral Mucosal and Salivary Microbial Community of Behcet's Syndrome and Recurrent Aphthous Stomatitis. J Oral Microbiol (2015) 7:27150. doi: 10.3402/jom.v7.27150 26037240 PMC4452653

[53] Mizushima Y Matsuda T Hoshi K Ohno S. Induction of Behçet's Disease Symptoms After Dental Treatment and Streptococcal Antigen Skin Test. J Rheumatol (1988) 15(6):1029–30. 3418627

[54] Karacayli U Mumcu G Simsek I Pay S Kose O Erdem H. The Close Association Between Dental and Periodontal Treatments and Oral Ulcer Course in Behcet's Disease: A Prospective Clinical Study. J Oral Pathol Med (2009) 38(5):410–5. doi: 10.1111/j.1600-0714.2009.00765.x 19320802

[55] Mumcu G Niazi S Stewart J Hagi-Pavli E Gokani B Seoudi N. Oral Health and Related Quality of Life Status in Patients From UK and Turkey: A Comparative Study in Behcet's Disease. J Oral Pathol Med (2009) 38(5):406–9. doi: 10.1111/j.1600-0714.2009.00752.x 19298505

[56] Akman A Kacaroglu H Donmez L Bacanli A Alpsoy E. Relationship Between Periodontal Findings and Behçet's Disease: A Controlled Study. J Clin Periodontol (2007) 34(6):485–91. doi: 10.1111/j.1600-051X.2007.01085.x 17451414

[57] Celenligil-Nazliel H Kansu E Ebersole JL. Periodontal Findings and Systemic Antibody Responses to Oral Microorganisms in Behçet's Disease. J Periodontol (1999) 70(12):1449–56. doi: 10.1902/jop.1999.70.12.1449 10632520

[58] Mumcu G Fortune F. Oral Health and Its Aetiological Role in Behçet's Disease. Front Med (Lausanne) (2021) 8:613419. doi: 10.3389/fmed.2021.613419 34095159 PMC8172597

[59] Lehner T. Immunological Aspects of Recurrent Oral Ulceration and Behcet's Syndrome. J Oral Pathol (1978) 7(6):424–30. doi: 10.1111/j.1600-0714.1978.tb01613.x 105103

[60] Kose O Stewart J Waseem A Lalli A Fortune F. Expression of Cytokeratins, Adhesion and Activation Molecules in Oral Ulcers of Behcet's Disease. Clin Exp Dermatol (2008) 33(1):62–9. doi: 10.1111/j.1365-2230.2007.02558.x 17983454

[61] Natah SS Konttinen YT Enattah NS Ashammakhi N Sharkey KA Häyrinen-Immonen R. Recurrent Aphthous Ulcers Today: A Review of the Growing Knowledge. Int J Oral Maxillofac Surg (2004) 33(3):221–34. doi: 10.1006/ijom.2002.0446 15287304

[62] Natah SS Hayrinen-Immonen R Hietanen J Patinen P Malmstrom M Savilahti E. Increased Density of Lymphocytes Bearing Gamma/Delta T-Cell Receptors in Recurrent Aphthous Ulceration (RAU). Int J Oral Maxillofac Surg (2000) 29(5):375–80. doi: 10.1034/j.1399-0020.2000.290514.x 11071244

[63] Pépin LF Roger T Morisset J Seman M. Preferential V Delta 1 Expression Among TcR Gamma/Delta-Bearing T Cells in Human Oral Epithelium. Scandinavian J Immunol (1993) 37(3):289–94. doi: 10.1111/j.1365-3083.1993.tb02556.x 8382839

[64] Dalghous AM Freysdottir J Fortune F. Expression of Cytokines, Chemokines, and Chemokine Receptors in Oral Ulcers of Patients With Behcet's Disease (BD) and Recurrent Aphthous Stomatitis Is Th1-Associated, Although Th2-Association Is Also Observed in Patients With BD. Scand J Rheumatol (2006) 35(6):472–5. doi: 10.1080/03009740600905380 17343257

[65] Wang X Dong L Liang Y Ni H Tang J Xu C. Performance Evaluation of FlowCytomix Assays to Quantify Cytokines in Patients With Rheumatoid Arthritis. Int J Clin Exp Med (2015) 8(9):16158–66. PMC4659018 26629129

[66] de Jager W Bourcier K Rijkers GT Prakken BJ Seyfert-Margolis V. Prerequisites for Cytokine Measurements in Clinical Trials With Multiplex Immunoassays. BMC Immunol (2009) 10:52–. doi: 10.1186/1471-2172-10-52 PMC2761376 19785746

[67] Burska A Boissinot M Ponchel F. Cytokines as Biomarkers in Rheumatoid Arthritis. Mediators Inflamm (2014) 2014:545493. doi: 10.1155/2014/545493 24733962 PMC3964841

[68] Mumcu G Cimilli H Karacayli U Inanc N Ture-Ozdemir F Eksioglu-Demiralp E. Salivary Levels of Antimicrobial Peptides Hnp 1-3, Ll-37 and S100 in Behcet's Disease. Arch Oral Biol (2012) 57(6):642–6. doi: 10.1016/j.archoralbio.2011.11.003 22153317

[69] Mumcu G Inanç N Özdemir FT Tulunay A Ekşioğlu-Demiralp E Ergun T. Effects of Azithromycin on Intracellular Cytokine Responses and Mucocutaneous Manifestations in Behçet's Disease. Int J Dermatol (2013) 52(12):1561–6. doi: 10.1111/ijd.12144 23879671

[70] Kucukkolbasi H Kucukkolbasi S Ayyildiz HF Dursun R Kara H. Evaluation of hbetaD-1 and hbetaD-2 Levels in Saliva of Patients With Oral Mucosal Diseases. West Indian Med J (2013) 62(3):230–8. 24564045

[71] Adişen E Aral A Aybay C Gürer MA. Salivary Epidermal Growth Factor Levels in Behçet's Disease and Recurrent Aphthous Stomatitis. Dermatology (2008) 217(3):235–40. doi: 10.1159/000148250 18663306

[72] Yoshizawa JM Schafer CA Schafer JJ Farrell JJ Paster BJ Wong DT. Salivary Biomarkers: Toward Future Clinical and Diagnostic Utilities. Clin Microbiol Rev (2013) 26(4):781–91. doi: 10.1128/CMR.00021-13 PMC3811231 24092855

[73] Qin R Steel A Fazel N. Oral Mucosa Biology and Salivary Biomarkers. Clin Dermatol (2017) 35(5):477–83. doi: 10.1016/j.clindermatol.2017.06.005 28916029

[74] Dawes C Wong DTW. Role of Saliva and Salivary Diagnostics in the Advancement of Oral Health. J Dent Res (2019) 98(2):133–41. doi: 10.1177/0022034518816961 PMC6900436 30782091

[75] Melguizo-Rodríguez L Costela-Ruiz VJ Manzano-Moreno FJ Ruiz C Illescas-Montes R. Salivary Biomarkers and Their Application in the Diagnosis and Monitoring of the Most Common Oral Pathologies. Int J Mol Sci (2020) 21(14):1–17. doi: 10.3390/ijms21145173 PMC7403990 32708341

[76] Konkel JE O'Boyle C Krishnan S. Distal Consequences of Oral Inflammation. Front Immunol (2019) 10:1403. doi: 10.3389/fimmu.2019.01403 31293577 PMC6603141

[77] Groeger S Meyle J. Oral Mucosal Epithelial Cells. Front Immunol (2019) 10:208. doi: 10.3389/fimmu.2019.00208 30837987 PMC6383680

[78] Corthay A. A Three-Cell Model for Activation of Naïve T Helper Cells. Scand J Immunol (2006) 64(2):93–6. doi: 10.1111/j.1365-3083.2006.01782.x 16867153

[79] Cai B Cai JP Luo YL Chen C Zhang S. The Specific Roles of JAK/STAT Signaling Pathway in Sepsis. Inflammation (2015) 38(4):1599–608. doi: 10.1007/s10753-015-0135-z 25676437

[80] Zouboulis CC Katsantonis J Ketteler R Treudler R Kaklamani E Hornemann S. Adamantiades-Behçet's Disease: Interleukin-8 Is Increased in Serum of Patients With Active Oral and Neurological Manifestations and Is Secreted by Small Vessel Endothelial Cells. Arch Dermatol Res (2000) 292(6):279–84. doi: 10.1007/s004030000128 10929768

[81] Sahin S Akoğlu T Direskeneli H Sen LS Lawrence R. Neutrophil Adhesion to Endothelial Cells and Factors Affecting Adhesion in Patients With Behçet's Disease. Ann Rheum Dis (1996) 55(2):128–33. doi: 10.1136/ard.55.2.128 PMC1010107 8712863

[82] Durmazlar SP Ulkar GB Eskioglu F Tatlican S Mert A Akgul A. Significance of Serum Interleukin-8 Levels in Patients With Behcet's Disease: High Levels may Indicate Vascular Involvement. Int J Dermatol (2009) 48(3):259–64. doi: 10.1111/j.1365-4632.2009.03905.x 19261013

[83] Alpsoy E Kodelja V Goerdt S Orfanos CE Zouboulis CC. Serum of Patients With Behçet's Disease Induces Classical (Pro-Inflammatory) Activation of Human Macrophages *In Vitro* . Dermatology (2003) 206(3):225–32. doi: 10.1159/000068888 12673080

[84] Mantas C Direskeneli H Oz D Yavuz S Akoglu T. IL-8 Producing Cells in Patients With Behçet's Disease. Clin Exp Rheumatol (2000) 18(2):249–51. 10812500

[85] Murphy PM Tiffany HL. Cloning of Complementary DNA Encoding a Functional Human Interleukin-8 Receptor. Science (1991) 253(5025):1280–3. doi: 10.1126/science.1891716 1891716

[86] Morohashi H Miyawaki T Nomura H Kuno K Murakami S Matsushima K. Expression of Both Types of Human Interleukin-8 Receptors on Mature Neutrophils, Monocytes, and Natural Killer Cells. J Leukoc Biol (1995) 57(1):180–7. doi: 10.1002/jlb.57.1.180 7829970

[87] Dinarello CA. Biologic Basis for Interleukin-1 in Disease. Blood (1996) 87(6):2095–147. doi: 10.1182/blood.V87.6.2095.bloodjournal8762095 8630372

[88] Cauci S Guaschino S De Aloysio D Driussi S De Santo D Penacchioni P. Interrelationships of Interleukin-8 With Interleukin-1beta and Neutrophils in Vaginal Fluid of Healthy and Bacterial Vaginosis Positive Women. Mol Hum Reprod (2003) 9(1):53–8. doi: 10.1093/molehr/gag003 12529421

[89] Düzgün N Ayaşlioğlu E Tutkak H Aydintuğ OT. Cytokine Inhibitors: Soluble Tumor Necrosis Factor Receptor 1 and Interleukin-1 Receptor Antagonist in Behçet's Disease. Rheumatol Int (2005) 25(1):1–5. doi: 10.1007/s00296-003-0400-6 14600787

[90] Dinarello CA. The IL-1 Family and Inflammatory Diseases. Clin Exp Rheumatol (2002) 20(5 Suppl 27):S1–13. 14989423

[91] Katsantonis J Adler Y Orfanos CE Zouboulis CC. Adamantiades-Behçet's Disease: Serum IL-8 Is a More Reliable Marker for Disease Activity Than C-Reactive Protein and Erythrocyte Sedimentation Rate. Dermatology (2000) 201(1):37–9. doi: 10.1159/000018426 10971057

[92] Schueller K Riva A Pfeiffer S Berry D Somoza V. Members of the Oral Microbiota Are Associated With IL-8 Release by Gingival Epithelial Cells in Healthy Individuals. Front Microbiol (2017) 8:416. doi: 10.3389/fmicb.2017.00416 28360899 PMC5350107

[93] Kashiwagi Y Yanagita M Kojima Y Shimabukuro Y Murakami S. Nicotine Up-Regulates IL-8 Expression in Human Gingival Epithelial Cells Following Stimulation With IL-1β or P. Gingivalis Lipopolysaccharide *via* Nicotinic Acetylcholine Receptor Signalling. Arch Oral Biol (2012) 57(5):483–90. doi: 10.1016/j.archoralbio.2011.10.007 22119045

[94] Nasser MW Raghuwanshi SK Grant DJ Jala VR Rajarathnam K Richardson RM. Differential Activation and Regulation of CXCR1 and CXCR2 by CXCL8 Monomer and Dimer. J Immunol (2009) 183(5):3425–32. doi: 10.4049/jimmunol.0900305 PMC2860795 19667085

[95] Hannig C Hannig M Kensche A Carpenter G. The Mucosal Pellicle - An Underestimated Factor in Oral Physiology. Arch Oral Biol (2017) 80:144–52. doi: 10.1016/j.archoralbio.2017.04.001 28419912

[96] Lee S Margolin K. Cytokines in Cancer Immunotherapy. Cancers (Basel) (2011) 3(4):3856–93. doi: 10.3390/cancers3043856 PMC3763400 24213115

[97] Schroder K Hertzog PJ Ravasi T Hume DA. Interferon-Gamma: An Overview of Signals, Mechanisms and Functions. J Leukoc Biol (2004) 75(2):163–89. doi: 10.1189/jlb.0603252 14525967

[98] Young HA Bream JH. IFN-Gamma: Recent Advances in Understanding Regulation of Expression, Biological Functions, and Clinical Applications. Curr Top Microbiol Immunol (2007) 316:97–117. doi: 10.1007/978-3-540-71329-6_6 17969445

[99] Aridogan BC Yildirim M Baysal V Inaloz HS Baz K Kaya S. Serum Levels of IL-4, IL-10, IL-12, IL-13 and IFN-Gamma in Behçet's Disease. J Dermatol (2003) 30(8):602–7. doi: 10.1111/j.1346-8138.2003.tb00442.x 12928529

[100] Tracey KJ Fong Y Hesse DG Manogue KR Lee AT Kuo GC. Anti-Cachectin/TNF Monoclonal Antibodies Prevent Septic Shock During Lethal Bacteraemia. Nature (1987) 330(6149):662–4. doi: 10.1038/330662a0 3317066

[101] Feldmann M Brennan FM Foxwell BM Maini RN. The Role of TNF Alpha and IL-1 in Rheumatoid Arthritis. Curr Dir Autoimmun (2001) 3:188–99. doi: 10.1159/000060522 11791466

[102] Formanek M Knerer B Kornfehl J. Cytokine Expression of Human Oral Keratinocytes. ORL J Otorhinolaryngol Relat Spec (1999) 61(2):103–7. doi: 10.1159/000027650 10095201

[103] Xu Q Izumi K Tobita T Nakanishi Y Feinberg SE. Constitutive Release of Cytokines by Human Oral Keratinocytes in an Organotypic Culture. J Oral Maxillofac Surg (2009) 67(6):1256–64. doi: 10.1016/j.joms.2009.02.003 PMC2727554 19446213

[104] Ozdamar Y Berker N Bahar G Soykan E Bicer T Ozkan SS. Inflammatory Mediators and Posterior Segment Involvement in Ocular Behcet Disease. Eur J Ophthalmol (2009) 19(6):998–1003. doi: 10.1177/112067210901900616 19882576

[105] Hatemi G Silman A Bang D Bodaghi B Chamberlain AM Gul A. Management of Behçet Disease: A Systematic Literature Review for the European League Against Rheumatism Evidence-Based Recommendations for the Management of Behçet Disease. Ann Rheum Dis (2009) 68(10):1528–34. doi: 10.1136/ard.2008.087957 18420940

[106] Robertson LP Hickling P. Treatment of Recalcitrant Orogenital Ulceration of Behçet's Syndrome With Infliximab. Rheumatol (Oxford) (2001) 40(4):473–4. doi: 10.1093/rheumatology/40.4.473 11312390

[107] Taylor LJ Bagg J Walker DM Peters TJ. Increased Production of Tumour Necrosis Factor by Peripheral Blood Leukocytes in Patients With Recurrent Oral Aphthous Ulceration. J Oral Pathol Med (1992) 21(1):21–5. doi: 10.1111/j.1600-0714.1992.tb00963.x 1593490

[108] Sun L He C Nair L Yeung J Egwuagu CE. Interleukin 12 (IL-12) Family Cytokines: Role in Immune Pathogenesis and Treatment of CNS Autoimmune Disease. Cytokine (2015) 75(2):249–55. doi: 10.1016/j.cyto.2015.01.030 PMC4553122 25796985

[109] Whittall T Wang Y Kelly CG Thompson R Sanderson J Lomer M. Tumour Necrosis Factor-Alpha Production Stimulated by Heat Shock Protein 70 and Its Inhibition in Circulating Dendritic Cells and Cells Eluted From Mucosal Tissues in Crohn's Disease. Clin Exp Immunol (2006) 143(3):550–9. doi: 10.1111/j.1365-2249.2006.03010.x PMC1809607 16487255

[110] Sadeghi A Davatchi F Shahram F Karimimoghadam A Alikhani M Pezeshgi A. Serum Profiles of Cytokines in Behcet's Disease. J Clin Med (2017) 6(5):49. doi: 10.3390/jcm6050049 PMC5447940 28468317

[111] Gholijani N Ataollahi MR Samiei A Aflaki E Shenavandeh S Kamali-Sarvestani E. An Elevated Pro-Inflammatory Cytokines Profile in Behcet's Disease: A Multiplex Analysis. Immunol Lett (2017) 186:46–51. doi: 10.1016/j.imlet.2016.12.001 27939191

[112] Naka T Nishimoto N Kishimoto T. The Paradigm of IL-6: From Basic Science to Medicine. Arthritis Res (2002) 4 Suppl 3(Suppl 3):S233–42. doi: 10.1186/ar565 PMC3240141 12110143

[113] Busse D de la Rosa M Hobiger K Thurley K Flossdorf M Scheffold A. Competing Feedback Loops Shape IL-2 Signaling Between Helper and Regulatory T Lymphocytes in Cellular Microenvironments. Proc Natl Acad Sci USA (2010) 107(7):3058–63. doi: 10.1073/pnas.0812851107 PMC2840293 20133667

[114] Yamakawa Y Sugita Y Nagatani T Takahashi S Yamakawa T Tanaka S. Interleukin-6 (IL-6) in Patients With Behçet's Disease. J Dermatol Sci (1996) 11(3):189–95. doi: 10.1016/0923-1811(95)00439-4 8785169

[115] Hirano T. Interleukin 6 in Autoimmune and Inflammatory Diseases: A Personal Memoir. Proc Jpn Acad Ser B Phys Biol Sci (2010) 86(7):717–30. doi: 10.2183/pjab.86.717 PMC3066534 20689230

[116] Shimizu J Takai K Fujiwara N Arimitsu N Ueda Y Wakisaka S. Excessive CD4+ T Cells Co-Expressing Interleukin-17 and Interferon-γ in Patients With Behçet's Disease. Clin Exp Immunol (2012) 168(1):68–74. doi: 10.1111/j.1365-2249.2011.04543.x 22385240 PMC3390496

[117] Chi W Zhu X Yang P Liu X Lin X Zhou H. Upregulated IL-23 and IL-17 in Behçet Patients With Active Uveitis. Invest Ophthalmol Vis Sci (2008) 49(7):3058–64. doi: 10.1167/iovs.07-1390 18579762

[118] Miossec P. Local and Systemic Effects of IL-17 in Joint Inflammation: A Historical Perspective From Discovery to Targeting. Cell Mol Immunol (2021) 18(4):860–5. doi: 10.1038/s41423-021-00644-5 PMC7943939 33692481

[119] Lubberts E. The IL-23-IL-17 Axis in Inflammatory Arthritis. Nat Rev Rheumatol (2015) 11(7):415–29. doi: 10.1038/nrrheum.2015.53 25907700

[120] Raifer H Mahiny AJ Bollig N Petermann F Hellhund A Kellner K. Unlike αβ T Cells, γδ T Cells, LTi Cells and NKT Cells Do Not Require IRF4 for the Production of IL-17A and IL-22. Eur J Immunol (2012) 42(12):3189–201. doi: 10.1002/eji.201142155 22961652

[121] Alunno A Bistoni O Bartoloni E Caterbi S Bigerna B Tabarrini A. IL-17-Producing CD4-CD8- T Cells Are Expanded in the Peripheral Blood, Infiltrate Salivary Glands and Are Resistant to Corticosteroids in Patients With Primary Sjögren's Syndrome. Ann Rheumatic Dis (2012) 72:286–92. doi: 10.1136/annrheumdis-2012-201511 22904262

[122] Ambrosi A Espinosa A Wahren-Herlenius M. IL-17: A New Actor in IFN-Driven Systemic Autoimmune Diseases. Eur J Immunol (2012) 42(9):2274–84. doi: 10.1002/eji.201242653 22949326

[123] Mellett M Atzei P Horgan A Hams E Floss T Wurst W. Orphan Receptor IL-17RD Tunes IL-17A Signalling and Is Required for Neutrophilia. Nat Commun (2012) 3:1119. doi: 10.1038/ncomms2127 23047677

[124] Hajishengallis G. New Developments in Neutrophil Biology and Periodontitis. Periodontol 2000 (2020) 82(1):78–92. doi: 10.1111/prd12313 31850633

[125] Ozçaka O Biçakci N Pussinen P Sorsa T Köse T Buduneli N. Smoking and Matrix Metalloproteinases, Neutrophil Elastase and Myeloperoxidase in Chronic Periodontitis. Oral Dis (2011) 17(1):68–76. doi: 10.1111/j.1601-0825.2010.01705.x 20646231

[126] Shen H Xia LP Lu J. Elevated Levels of Interleukin-27 and Effect on Production of Interferon-γ and Interleukin-17 in Patients With Behçet's Disease. Scand J Rheumatol (2013) 42(1):48–51. doi: 10.3109/03009742.2012.704391 23101722

[127] Curnow SJ Pryce K Modi N Knight B Graham EM Stewart JE. Serum Cytokine Profiles in Behçet's Disease: Is There a Role for IL-15 in Pathogenesis? Immunol Lett (2008) 121(1):7–12. doi: 10.1016/j.imlet.2008.07.009 18706446

[128] Szpaderska AM Zuckerman JD DiPietro LA. Differential Injury Responses in Oral Mucosal and Cutaneous Wounds. J Dent Res (2003) 82(8):621–6. doi: 10.1177/154405910308200810 12885847

[129] Irwin CR Myrillas TT. The Role of IL-6 in the Pathogenesis of Periodontal Disease. Oral Dis (1998) 4(1):43–7. doi: 10.1111/j.1601-0825.1998.tb00255.x 9655045

[130] Dutzan N Abusleme L Bridgeman H Greenwell-Wild T Zangerle-Murray T Fife ME. On-Going Mechanical Damage From Mastication Drives Homeostatic Th17 Cell Responses at the Oral Barrier. Immunity (2017) 46(1):133–47. doi: 10.1016/j.immuni.2016.12.010 PMC5263257 28087239

[131] Choi P Reiser H. IL-4: Role in Disease and Regulation of Production. Clin Exp Immunol (1998) 113(3):317–9. doi: 10.1046/j.1365-2249.1998.00690.x PMC1905061 9737656

[132] Peranteau WH Zhang L Muvarak N Badillo AT Radu A Zoltick PW. IL-10 Overexpression Decreases Inflammatory Mediators and Promotes Regenerative Healing in an Adult Model of Scar Formation. J Invest Dermatol (2008) 128(7):1852–60. doi: 10.1038/sj.jid.5701232 18200061

[133] Couper KN Blount DG Riley EM. IL-10: The Master Regulator of Immunity to Infection. J Immunol (2008) 180(9):5771–7. doi: 10.4049/jimmunol.180.9.5771 18424693

[134] Turan B Gallati H Erdi H Gürler A Michel BA Villiger PM. Systemic Levels of the T Cell Regulatory Cytokines IL-10 and IL-12 in Bechçet's Disease; Soluble TNFR-75 as a Biological Marker of Disease Activity. J Rheumatol (1997) 24(1):128–32. 9002023

[135] Romagnani S. Biology of Human TH1 and TH2 Cells. J Clin Immunol (1995) 15(3):121–9. doi: 10.1007/BF01543103 7559914

[136] Marques CP Victor EC Franco MM Fernandes JM Maor Y de Andrade MS. Salivary Levels of Inflammatory Cytokines and Their Association to Periodontal Disease in Systemic Lupus Erythematosus Patients. A Case-Control Study Cytokine (2016) 85:165–70. doi: 10.1016/j.cyto.2016.06.025 27371775

[137] Chiamulera MMA Zancan CB Remor AP Cordeiro MF Gleber-Netto FO Baptistella AR. Salivary Cytokines as Biomarkers of Oral Cancer: A Systematic Review and Meta-Analysis. BMC Cancer (2021) 21(1):205. doi: 10.1186/s12885-021-07932-3 33639868 PMC7912500

[138] Ter Horst R Jaeger M Smeekens SP Oosting M Swertz MA Li Y. Host and Environmental Factors Influencing Individual Human Cytokine Responses. Cell (2016) 167(4):1111–24.e13. doi: 10.1016/j.cell.2016.10.018 27814508 PMC5787854

[139] Li Y Oosting M Smeekens SP Jaeger M Aguirre-Gamboa R Le KTT. A Functional Genomics Approach to Understand Variation in Cytokine Production in Humans. Cell (2016) 167(4):1099–110.e14. doi: 10.1016/j.cell.2016.10.017 27814507

[140] Ter Horst R Jaeger M van de Wijer L van der Heijden WA Janssen AMW Smeekens SP. Seasonal and Nonseasonal Longitudinal Variation of Immune Function. J Immunol (2021) 207:696–708. doi: 10.4049/jimmunol.2000133 34261668

[141] Doumas BT Bayse DD Carter RJ Peters T Jr. Schaffer R. A Candidate Reference Method for Determination of Total Protein in Serum. I. Development and Validation. Clin Chem (1981) 27(10):1642–50. doi: 10.1093/clinchem/27.10.1642 6169466

[142] Hori Y Nishida K Yamato M Sugiyama H Soma T Inoue T. Differential Expression of MUC16 in Human Oral Mucosal Epithelium and Cultivated Epithelial Sheets. Exp Eye Res (2008) 87(3):191–6. doi: 10.1016/j.exer.2008.05.014 18644592

[143] Turner MD Nedjai B Hurst T Pennington DJ. Cytokines and Chemokines: At the Crossroads of Cell Signalling and Inflammatory Disease. Biochim Biophys Acta (2014) 1843(11):2563–82. doi: 10.1016/j.bbamcr.2014.05.014 24892271

