# Supplementary material for: Saliva and Serum Cytokine Profiles During Oral Ulceration in Behçet’s Disease
Source: Front Immunol. 2021 Dec 22;12:724900. doi: 10.3389/fimmu.2021.724900 (PMC8727526; doi:10.3389/fimmu.2021.724900)
Supplement: Supplementary file 1 [file DataSheet_1.pdf]

ΔCytokine Concentrations (pg/ml) ($BD_{conc.}$-$HC_{conc.}$ or $RAS_{conc.}$-$HC_{conc.}$)				
	ΔBDsaliva N=20	ΔBDserum N=20	ΔRASsaliva N=7	ΔRASserum N=7
IL-1β	736.6	0.00	1056.6	0.00
IL-2	-11.4	20.55	-49.7	59.60
IL-4	-3.48	0.00	-18.73	0.00
IL-5	-18.14	0.00	-23.16	0.00
IL-6	7.59	0.00	5.71	0.00
IL-8	178.2	17.6	575.7	-63.19
IL-10	20.35	11.92	40.3	0.00
IL-12p70	-28.09	0.00	80.10	0.00
IL-17A	1.29	0.00	3.24	0.00
IFN-γ	0.00	0.00	0.00	0.00
TNF-α	15.57	0.94	1.97	-4.57
TNF-β	5.52	0.00	59.99	0.00

Table S1. Calculating the median cytokine level differences between each disease group and HC baseline. For each cytokine, the following was calculated: Group Median BD cytokine concentration (pg/ml) - Group Median HC cytokine concentration (pg/ml) or Group Median RAS cytokine concentration (pg/ml) - Group Median HC cytokine concentration (pg/ml) to reveal the delta, or difference, of the disease group cytokine levels and the HC baseline. Data is represented in Figure 2A and 2B.

	Saliva Concentration (pg/ml) (IQR)			Saliva Ratios			Serum Concentration (pg/ml) (IQR)			Serum Ratios		
	BD	RAS	HC	BD/HC	RAS/HC	BD/RAS	BD	RAS	HC	BD/HC	RAS/HC	BD/RAS
IL-1β	1489 (727.4-2383)	1809 (251.1-2399)	752.4 (447.1-1775)	1.97	2.40	-1.22	0.01 (0.01-36.81)	0.01 (0.01-10.03)	0.01 (0.01-0.01)	0	0	0
IL-2	176.4 (139.6-232.4)	138.1 (94.98-182.2)	187.8 (125.4-249.8)	-1.06	-1.35	1.28	40.58 (0.01-71.25)	79.63 (0.01-131.9)	20.3 (0.01-185.1)	2	3.9	-2
IL-4	91.36 (53.02-102.5)	76.11 (67.11-89.61)	94.84 (40.52-124.7)	-1.04	-1.25	1.20	0.01 (0.01-51.87)	0.01 (0.01-0.01)	0.01 (0.01-0.01)	0	0	0
IL-5	67.77 (51.34-117.5)	62.75 (46.87-212.6)	85.91 (36.86-119.1)	-1.25	-1.37	1.08	0.01 (0.01-13.5)	0.01 (0.01-32.81)	0.01 (0.01-21.23)	0	0	0
IL-6	17.05 (0.01-91.26)	15.17 (1.07-75.7)	9.46 (4.89-26.27)	1.8	1.6	1.12	0.01 (0.01-0.01)	0.01 (0.01-0.01)	0.01 (0.01-0.01)	0	0	0
IL-8	588.5 (329.6-1804)	986 (263.9-1744)	410.3 (237.5-966.9)	1.4	2.4	1.67	145 (72.6-535)	64.21 (12.08-162.8)	127.4 (25.06-975.5)	1.1	0.5	2.3
IL-10	70.83 (28.54-15.01)	90.78 (29.48-178.7)	50.48 (29.83-78.37)	1.4	1.8	-1.28	11.93 (0.01-16.46)	0.01 (0.01-0.01)	0.01 (0.01-12.76)	12	0	12
IL-12p70	88.41 (23.39-182.5)	196.6 (124.5-223.5)	116.5 (57.84-203.7)	-1.32	1.7	-2.22	0.01 (0.01-0.01)	0.01 (0.01-0.01)	0.01 (0.01-3.88)	0	0	0
IL-17A	25.67 (13.98-55.19)	27.62 (2.02-43.31)	24.38 (1.52-69.96)	1.05	1.13	-1.08	0.01 (0.01-0.01)	0.01 (0.01-0.01)	0.01 (0.01-5.8)	0	0	0
IFN-γ	0.01 (0.01-93.49)	0.01 (0.01-113.7)	0.01 (0.01-98.76)	0	0	0	0.01 (0.01-0.01)	0.01 (0.01-0.01)	0.01 (0.01-0.01)	0	0	0
TNF-α	55.81 (25.36-83.27)	42.21 (30.22-44.26)	40.24 (24.39-64.49)	1.40	1.05	1.32	5.52 (1.72-24.39)	0.01 (0.01-13.95)	4.58 (0.01-24.55)	1.2	0	5.5
TNF-β	92.93 (38.77-169.5)	147.4 (117.2-196.7)	87.41 (33.29-209)	1.06	1.67	-1.59	0.01 (0.01-0.01)	0.01 (0.01-0.01)	0.01 (0.01-32.13)	0	0	0

Supplementary Table S2. Concentrations of cytokines in saliva from BD N=20, RAS N=7 and HC N=10 and serum BD N=19, RAS N=7 and HC N=10. Median concentrations with interquartile ranges are shown in brackets. Cytokines below the lower levels limits of detection (see Table 2) were considered not detectable and therefore arbitrarily assigned concentrations of 0.01 pg/ml. Ratios between cytokine concentrations in saliva and serum are reported for: BD and HC, RAS and HC, where negative values indicate a higher level of expression in HCs and finally, BD and RAS, where negative values indicate a higher level of expression in RAS. Log scale graphs of the concentrations of all 12 cytokines in serum and saliva are shown in **Figures 1A-D, Supplementary Figure S1 and Figure S2** to compare and illustrate the marked difference in serum and cytokine levels in study groups.

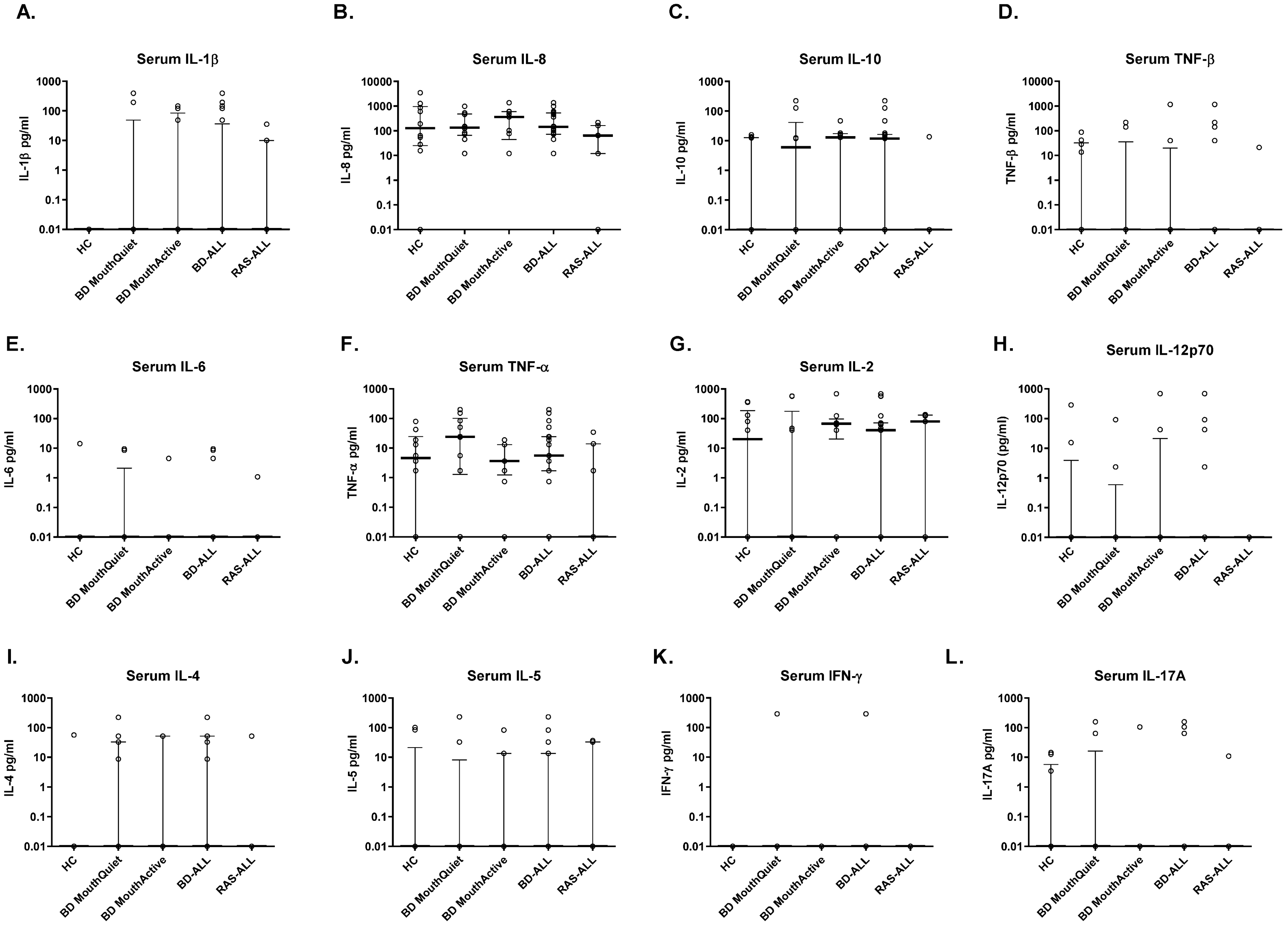

Figure S1. Serum cytokine levels. Plots show the cytokine concentrations (**pg/ml**) from BD-ALL (N=19*), RAS-ALL (N=7) and HC (N=10). The BD patients were further grouped into patients with oral ulceration, BD Mouth Active, BD-MA (N=9), and patients with no oral ulceration, BD Mouth Quiet, BD-MQ (N=10*). The median and interquartile range are shown (Median ± IQR). *Denotes that one BD-MQ serum sample was excluded from the analysis due to an erroneous flow cytometer reading

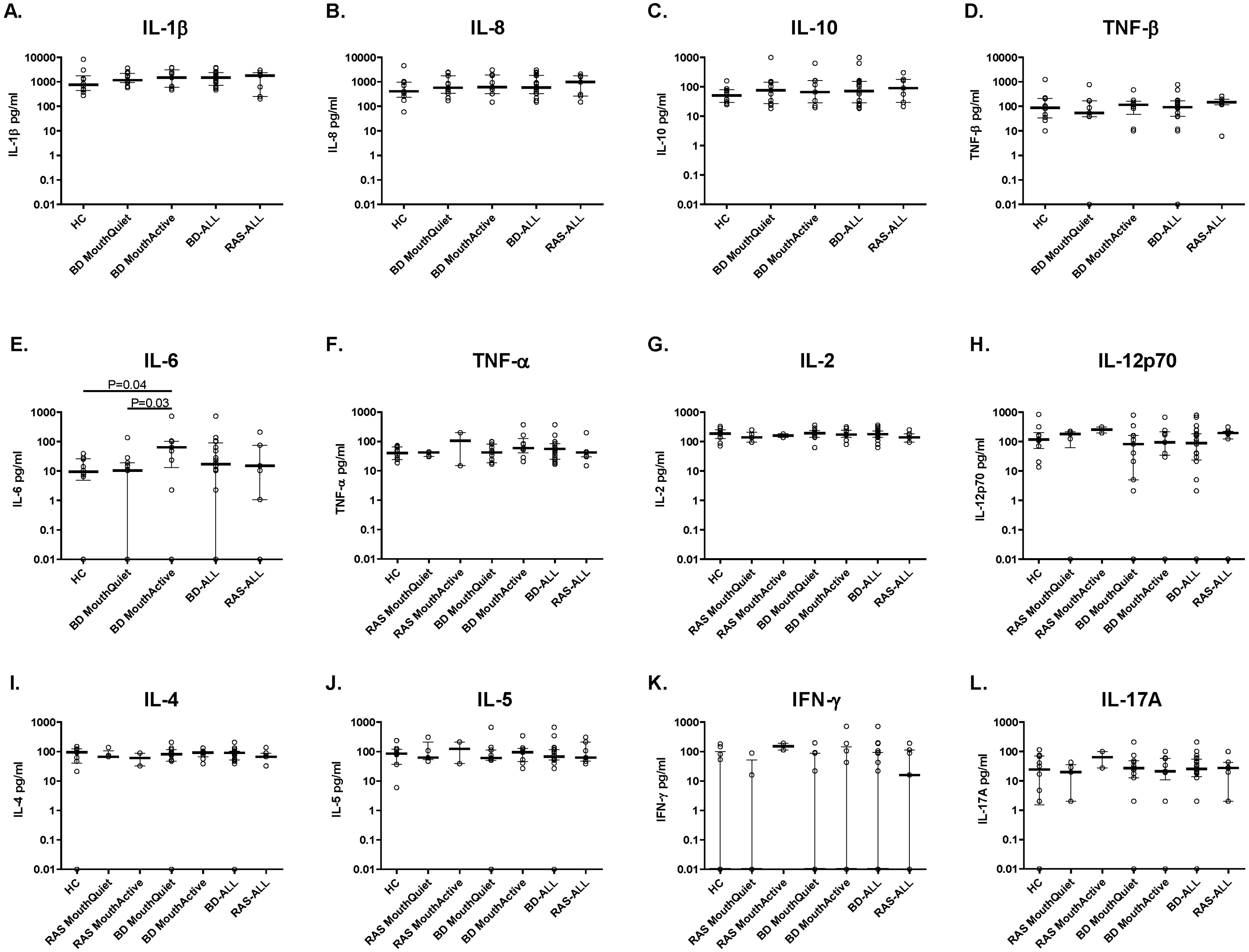

Figure S2 Saliva Cytokine levels differentially expressed in BD and RAS patients as compared to HCs. Plots show the cytokine concentrations (**pg/ml**) from BD-ALL (N=20), RAS-ALL (N=7) and HC (N=10). The BD patients were further grouped into patients with oral ulceration, BD Mouth Active, BD-MA (N=9), and patients with no oral ulceration, BD Mouth Quiet, BD-MQ (N=11). The median interquartile range are shown (Median ± IQR).

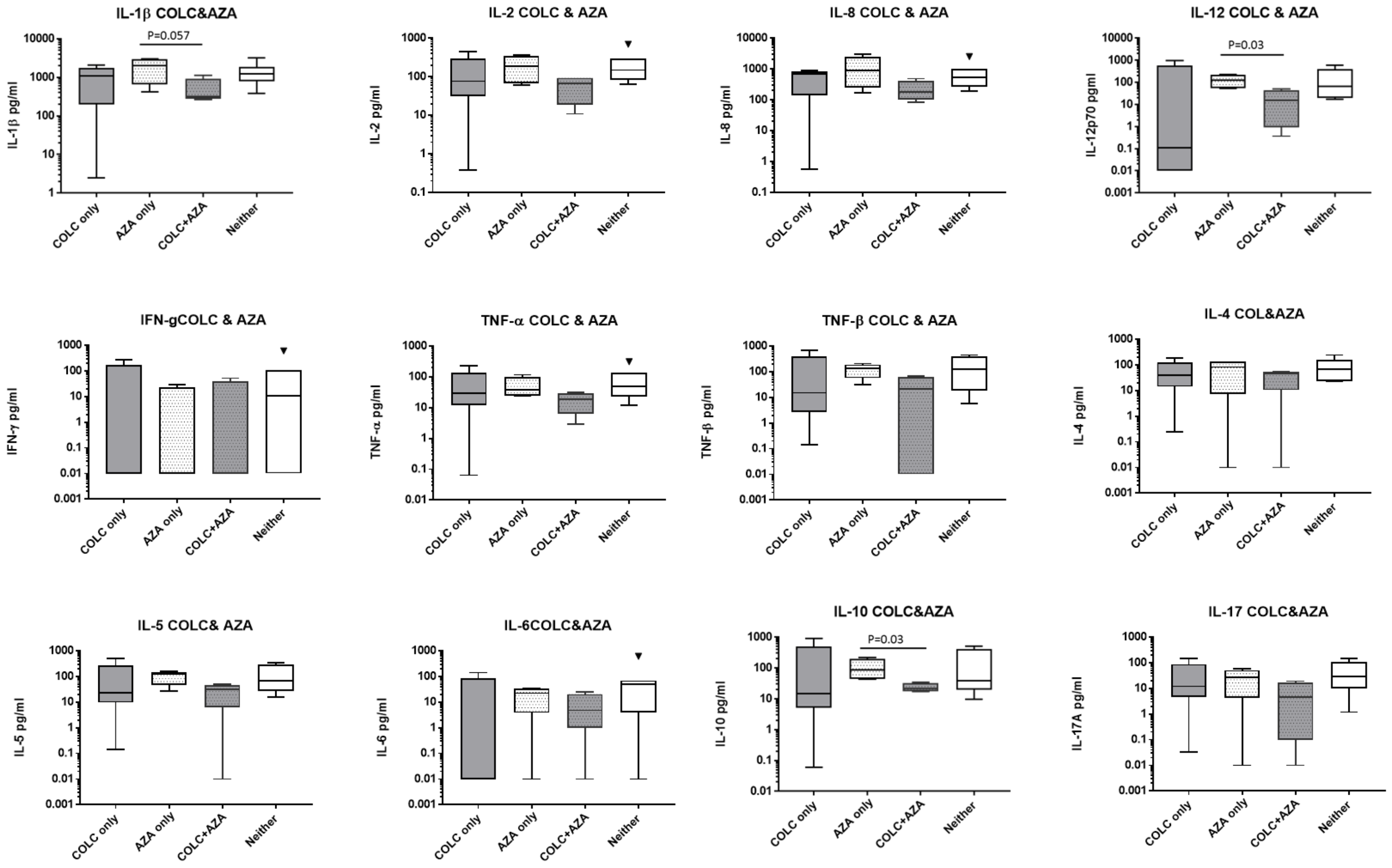

Figure S3. Effects of Medications: Normalised **Saliva** Cytokine concentrations in Patients (BD) taking colchicine (COLC) and/or Azathioprine (AZA). The Tukey box plots show levels in BD patients identified as taking COLC or AZA, both or neither AZA or COLC. COLC refers only to the exclusion of AZA and vice-versa, however this does not exclude other medications that the patient may have been taking. Mann-Whitney U test, significance based on two tailed 95% confidence interval (CI). Exact P values are indicated.

Cytokine analysed in Pilot study	Primary producing cells	Primary consuming cells	Key Reference
**IL-1β**	Monocytes, epithelial cells	Macrophages	Dinarello *et al* 1996
**IL-2**	$CD4^+$ T cells, NK cells	T_{reg} cells	Busse *et al* 2010
**IL-4**	T cells, NKT cells, γδ T cells, mast cells	B cells, T cells, macrophages	Corthay 2006
**IL-5**	T_H2 cells, mast cells, eosinophils, NK cells	B cells, eosinophils	Cai *et al* 2015
**IL-6**	T cells, macrophages, fibroblasts, endothelial cells	B cells, T cells, thymocytes	Cai *et al* 2015<br>Heinrich et al 1998
**IL-8**	Monocytes, T cells, neutrophils, fibroblasts, endothelial cells	Neutrophils	Cai *et al* 2015
**IL-10**	T_H2 cells, macrophages, DCs, B cells	T cells, macrophages	Couper *et al* 2008
**IL-12**	Activated macrophages, DCs	Activated T cells, NK cells	Sun et al 2015
**IL-17**	T_H17 cells, NK cells, NKT cells	Fibroblasts, endothelial cells, epithelial cells, keratinocytes, macrophages, DCs	Cai *et al* 2015<br>Miossec *et al* 2009
**IFNγ**	T cells, NK cells, NKT cells	T cells, monocytes, macrophages	Schroder *et al* 2004
**TNF**	T cells, B cells, NK cells, macrophages	T cells, B cells, endothelial cells	Lee and Margolin 2011

Abbreviated Example of the Cytokine Network

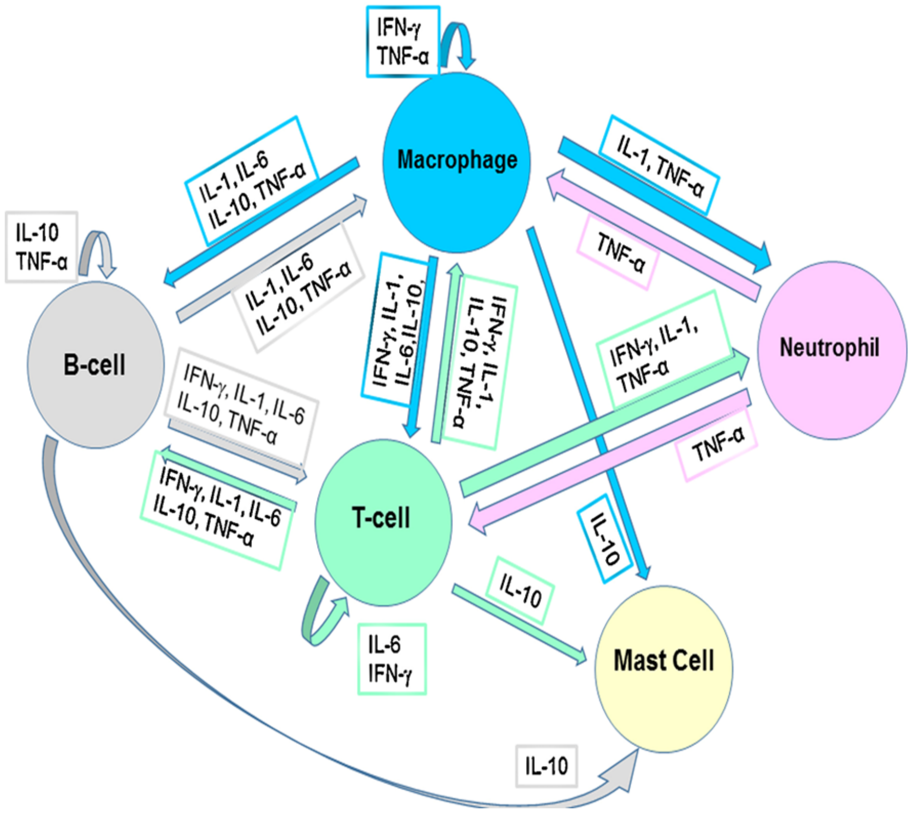

Adapted from Stenken and Poschenrieder 2015 with permission from the publishers.

Supplementary Figure S4: Cytokines used in the Pilot study showing Primary producer and consumer cells with an abbreviated example of cytokine networks. (Dinarello 1996)(Busse, de la Rosa et al. 2010)(Corthay 2006)(Cai, Cai et al. 2015)(Couper, Blount et al. 2008)(Sun, He et al. 2015)(Miossec, Korn et al. 2009)(Schroder, Hertzog et al. 2004)(Lee and Margolin 2011)

Busse, D., M. de la Rosa, K. Hobiger, K. Thurley, M. Flossdorf, A. Scheffold and T. Höfer (2010). "Competing feedback loops shape IL-2 signaling between helper and regulatory T lymphocytes in cellular microenvironments." Proc Natl Acad Sci U S A **107**(7): 3058-3063.
Cai, B., J. P. Cai, Y. L. Luo, C. Chen and S. Zhang (2015). "The Specific Roles of JAK/STAT Signaling Pathway in Sepsis." Inflammation **38**(4): 1599-1608.
Corthay, A. (2006). "A three-cell model for activation of naïve T helper cells." Scand J Immunol **64**(2): 93-96.
Couper, K. N., D. G. Blount and E. M. Riley (2008). "IL-10: the master regulator of immunity to infection." J Immunol **180**(9): 5771-5777.
Dinarello, C. A. (1996). "Biologic basis for interleukin-1 in disease." Blood **87**(6): 2095-2147.

Lee, S. and K. Margolin (2011). "Cytokines in cancer immunotherapy." Cancers (Basel) **3**(4): 3856-3893.
Miossec, P., T. Korn and V. K. Kuchroo (2009). "Interleukin-17 and type 17 helper T cells." N Engl J Med **361**(9): 888-898.
Schroder, K., P. J. Hertzog, T. Ravasi and D. A. Hume (2004). "Interferon-gamma: an overview of signals, mechanisms and functions." J Leukoc Biol **75**(2): 163-189.
Sun, L., C. He, L. Nair, J. Yeung and C. E. Egwuagu (2015). "Interleukin 12 (IL-12) family cytokines: Role in immune pathogenesis and treatment of CNS autoimmune disease." Cytokine **75**(2): 249-255.

Stenken, J.A. and Poschenrieder, A.J. (2015) ‘’ Bioanalytical Chemistry of Cytokines-A review.’’ Analytica Chimica Acta **853** : 95-115
